# Sex-Specific Relationship between the Cardiorespiratory Fitness and Plasma Metabolite Patterns in Healthy Humans—Results of the KarMeN Study

**DOI:** 10.3390/metabo11070463

**Published:** 2021-07-17

**Authors:** Sina Kistner, Maik Döring, Ralf Krüger, Manuela J. Rist, Christoph H. Weinert, Diana Bunzel, Benedikt Merz, Katrin Radloff, Rainer Neumann, Sascha Härtel, Achim Bub

**Affiliations:** 1 Department of Physiology and Biochemistry of Nutrition, Max Rubner-Institut, 76131 Karlsruhe, Germany; maik.doering@mri.bund.de (M.D.); ralf.krueger@mri.bund.de (R.K.); manuela.rist@mri.bund.de (M.J.R.); benedikt.merz@mri.bund.de (B.M.); k.radloff@pantherna-therapeutics.com (K.R.); achim.bub@kit.edu (A.B.); 2 Department of Safety and Quality of Fruit and Vegetables, Max Rubner-Institut, 76131 Karlsruhe, Germany; christoph.weinert@mri.bund.de (C.H.W.); diana.bunzel@mri.bund.de (D.B.); 3 Institute of Sports and Sports Science, Karlsruhe Institute of Technology, 76131 Karlsruhe, Germany; rainer.neumann@ph-karlsruhe.de (R.N.); sascha.haertel@achtzehn99.de (S.H.)

**Keywords:** cardiorespiratory fitness, physical fitness, metabolomics, plasma metabolome, plasma metabolite patterns, metabolite profiles

## Abstract

Cardiorespiratory fitness (CRF) represents a strong predictor of all-cause mortality and is strongly influenced by regular physical activity (PA). However, the biological mechanisms involved in the body’s adaptation to PA remain to be fully elucidated. The aim of this study was to systematically examine the relationship between CRF and plasma metabolite patterns in 252 healthy adults from the cross-sectional Karlsruhe Metabolomics and Nutrition (KarMeN) study. CRF was determined by measuring the peak oxygen uptake during incremental exercise. Fasting plasma samples were analyzed by nuclear magnetic resonance spectroscopy and mass spectrometry coupled to one- or two-dimensional gas chromatography or liquid chromatography. Based on this multi-platform metabolomics approach, 427 plasma analytes were detected. Bi- and multivariate association analyses, adjusted for age and menopausal status, showed that CRF was linked to specific sets of metabolites primarily indicative of lipid metabolism. However, CRF-related metabolite patterns largely differed between sexes. While several phosphatidylcholines were linked to CRF in females, single lyso-phosphatidylcholines and sphingomyelins were associated with CRF in males. When controlling for further assessed clinical and phenotypical parameters, sex-specific CRF tended to be correlated with a smaller number of metabolites linked to lipid, amino acid, or xenobiotics-related metabolism. Interestingly, sex-specific CRF explanation models could be improved when including selected plasma analytes in addition to clinical and phenotypical variables. In summary, this study revealed sex-related differences in CRF-associated plasma metabolite patterns and proved known associations between CRF and risk factors for cardiometabolic diseases such as fat mass, visceral adipose tissue mass, or blood triglycerides in metabolically healthy individuals. Our findings indicate that covariates like sex and, especially, body composition have to be considered when studying blood metabolic markers related to CRF.

## 1. Introduction

Cardiorespiratory fitness (CRF) is a health-related component of physical fitness (PF) [1], reflecting the ability of the circulatory, respiratory, and muscular systems to take up, transport, and utilize oxygen during sustained physical exercise (PE) [2]. It is affected by sex [3,4], age [4,5], lean body mass (LBM) [6], heredity [7], and behavioral factors like diet, smoking, or physical activity (PA) [4]. Actually, CRF is not only an objective measure of regular PA [8] but has also emerged as a strong predictor of all-cause and disease-specific mortality [9]. Representing the main modifiable determinant of CRF, regular PA is known to favorably influence body composition and glucose-insulin homeostasis, as well as the lipoprotein profile [8]. At the muscular level, beneficial adaptations to PA comprise an increased capillarization, a higher mitochondrial density, and enhanced oxidative metabolism, finally leading to an improved endurance capacity [10]. However, despite profound knowledge on the health-promoting benefits of PA, the molecular mechanisms and metabolic pathways involved in the whole-body and skeletal muscle adaptation to PA are still insufficiently understood [11].

The emerging field of metabolomics is a promising approach to systematically investigating exercise-induced changes in human metabolism related to performance and health [12]. By applying nuclear magnetic resonance (NMR) spectroscopy- or mass spectrometry (MS)-based techniques, metabolomics permits the simultaneous analysis of a high number and variety of metabolites, i.e., low-molecular-weight compounds, that represent the end-products of interactions between genes, proteins, and the cellular environment [13]. Thus, metabolomics can help to identify PA- or PF-associated metabolite profiles, possibly hinting at metabolic pathways that are linked to the well-known effects of exercise [12].

Interestingly, recent metabolomic studies have provided the first evidence that higher levels of PA or PF are linked to lower circulating branched-chain amino acid (AA) [14,15,16,17] and higher circulating phosphatidylcholine (PC) concentrations [18,19,20,21]. However, research on the relationship between CRF and the blood metabolome in large populations, including both sexes and with a broad age spectrum, is rather scarce. In fact, only half of the studies that assessed maximal oxygen uptake (VO_2max_) as the gold standard of aerobic fitness conducted correlation or regression analyses [17,19,20,22,23], while the other half examined differences between groups with high or low CRF [15,18,21,24]. Limitations of the former studies are that the results were restricted to young [17,22], middle-aged [19,20], or older [23] individuals and are thus hardly transferable to the general population. Besides, studies either had rather small sample sizes [22,23] or solely included male subjects [17]. Apart from Lustgarten et al., who detected nearly 300 serum analytes [22], the remaining studies focused on a limited number of metabolites. Since several CRF-associated metabolites that were reported in the literature have also been linked to other phenotypical variables such as body composition, adjustments for potential confounders are decisive to determine if correlations can be specifically attributed to CRF [25].

As a way of overcoming those limitations, we applied a multi-platform metabolomics approach and exploratory bi- and multivariate statistical procedures to systematically analyze the relationship between CRF and 427 plasma metabolites in 252 healthy women and men from the cross-sectional Karlsruhe Metabolomics and Nutrition (KarMeN) study. Participants had a wide age range and were thoroughly characterized by anthropometric, functional, and clinical examinations [26]. Therefore, we were able to take a variety of known and potential confounders into account. Since it has already been shown that sex, age, and menopausal status are determinants of CRF [4,27] and are also linked to a discriminatory plasma metabolite profile in the KarMeN participants [28], all analyses were conducted in sex-specific subgroups and adjusted for age and menopausal status. Firstly, both bi- and multivariate associations between CRF and metabolites were calculated, using bivariate correlation or partial least squares (PLS) regression analyses, respectively. Secondly, to identify associations that were independent of other phenotypical or clinical variables, correlation analyses and PLS models were additionally adjusted for parameters related to body composition, clinical blood biochemistry, lung and arterial function, short-term and habitual PA, or diet. Thirdly, cross-validated stepwise regression procedures were conducted, thus selecting sets of phenotypical, clinical, and plasma metabolite variables that contribute to a preferably good explanation of CRF.

## 2. Results

### 2.1. Metabolomics Data

427 plasma analytes were included in the final data analysis. Untargeted methods yielded 234 analytes, of which 43 (18.4%) could be identified with sufficient certainty. 193 analytes were derived from targeted analyses and were thus known a priori. Of the 236 identified metabolites, most belonged to lipid metabolism (69.5%) and AA metabolism (17.4%), followed by xenobiotics-related metabolism (5.1%), mammalian-microbial cometabolism (3.0%), carbohydrate or energy metabolism (each 2.1%), and nucleotide or cofactors and vitamins metabolism (each 0.4%). The classification of the identified metabolites to both major and specific metabolic pathways is provided in Table S1.

### 2.2. Basic Characteristics of Study Participants

**Figure 1:**
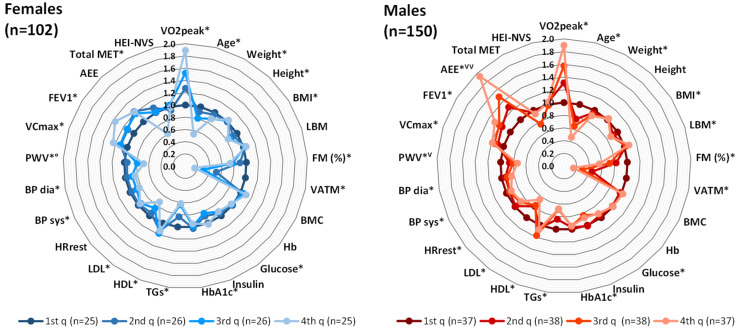
Radar plots visualizing the basic characteristics of KarMeN participants according to sex-specific VO_2peak_ quarters. The means of the 1st q were used as reference values to the means of the 2nd, 3rd and 4th q. *: significant differences between quarters according to the Welch ANOVA. °: n = 23 (1st q), n = 25 (2nd q); ⱽ: n = 36 (1st q); ⱽⱽ: n = 35 (1st q), n = 37 (2nd q). AEE: activity energy expenditure; BMC: bone mineral content; BMI: body mass index; BP: blood pressure; dia: diastolic; FEV1: forced expiratory pressure in one second; FM: fat mass; Hb: hemoglobin; HDL: high-density lipoprotein; HEI-NVS: Healthy Eating Index (modified version); HR_rest_: resting heart rate; LBM: lean body mass; LDL: low-density lipoprotein; MET: metabolic equivalent of task; PWV: pulse wave velocity; q: quarter; sys: systolic; TGs: triglycerides; VATM: visceral adipose tissue mass; VC_max_: maximal vital capacity; VO_2peak_: peak oxygen uptake.

The study sample consisted of 252 healthy individuals, 150 males (M, 59.5%) and 102 females (F, 40.5%), with a mean age of 45.9 ± 17.1 years and a mean peak oxygen uptake (VO_2peak_) of 38.9 ± 11.7 mL kg^−1^ min^−1^. The characteristics of participants are presented according to sex-specific VO_2peak_ quarters (Figure 1). In the radar plots, the respective means of the lowest VO_2peak_ quarter (1st q) were used as a reference value and the means of the other quarters (2nd q, 3rd q and 4th q) were related to the means of the first quarter. The absolute means, standard deviations, and respective units are provided in File S1.

In both females and males, there were statistically significant differences between VO_2peak_-related quarters with regard to age, weight, body mass index (BMI), fat mass (FM (%)), and visceral adipose tissue mass (VATM (in kg)). Furthermore, differences for clinical parameters like fasting blood glucose, HbA1c, triglycerides (TGs), high-density lipoprotein (HDL) and low-density lipoprotein (LDL) cholesterol, systolic and diastolic blood pressure (BP), pulse wave velocity (PWV), maximal vital capacity (VC_max_), and forced expiratory pressure in one second (FEV1) were observed across the quarters of the sex-specific VO_2peak_. In females, additional differences between VO_2peak_-related quarters could be observed for height and the total metabolic equivalent of task (MET), while in males, differences were detected for LBM, resting heart rate (HR_rest_), and the activity energy expenditure (AEE). The menopausal status in women also significantly differed between VO_2peak_ quarters, with increasing ratios of pre- to post-menopausal women from the 1st q to the 4th q (see File S1 (Table 1)). As the non-modifiable factors age and menopausal status were associated with the sex-specific VO_2peak_, and since they have already been shown to determine plasma metabolite patterns in KarMeN subjects [28], these variables were treated as confounders in all subsequent analyses.

### 2.3. Sex-Specific Relationship between CRF and Phenotypical/Clinical Variables

**Figure 2:**
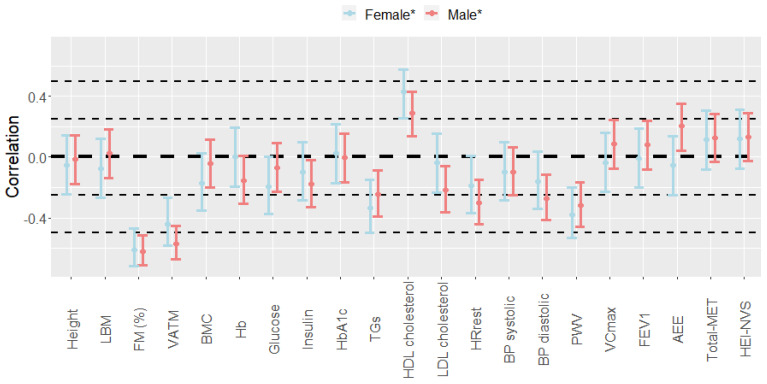
Confounder-adjusted sex-specific correlations between the VO_2peak_ and phenotypical/clinical variables. * Pearson correlations were performed on Van der Waerden (VdW)-transformed data adjusted for age (and menopausal status in females) and respective correlation coefficients (r; dots) and 95% confidence intervals (CIs; bars) are illustrated. AEE: activity energy expenditure; BMC: bone mineral content; BP: blood pressure; FEV1: forced expiratory pressure in one second; FM: fat mass; Hb: hemoglobin; HDL: high-density lipoprotein; HEI-NVS: Healthy Eating Index (modified version); HR_rest_: resting heart rate; LBM: lean body mass; LDL: low-density lipoprotein; MET: metabolic equivalent of task; PWV: pulse wave velocity; TGs: triglycerides; VATM: visceral adipose tissue mass; VC_max_: maximal vital capacity.

To examine sex-specific relations between the VO_2peak_ and selected phenotypical as well as clinical variables, correlations adjusted for age (and menopausal status in females) were calculated. A visual comparison of the pairwise correlations in women and men is provided in Figure 2. After adjustments for the above-mentioned confounding factors, the VO_2peak_ in females showed positive correlations with HDL cholesterol (r = 0.43) and negative correlations with the FM (%) (r = −0.61), VATM (r = −0.44), PWV (r = −0.38), and TGs (r = −0.33), that were all significantly different from zero. In males, not only HDL cholesterol (r = 0.29) but also the AEE (r = 0.20) showed positive correlations with the VO_2peak_. Compared to females, negative correlations to the VO_2peak_ that were significantly different from zero were not only detected for the FM (%) (r = −0.62), VATM (r = −0.57), PWV (r = −0.32), and TGs (r = −0.25) but additionally for HR_rest_ (r = −0.30), diastolic BP (r = −0.27), LDL cholesterol (r = −0.22) and insulin (r = −0.18).

### 2.4. Sex-Specific Relationship between CRF and Plasma Metabolites

#### 2.4.1. Bivariate Association Analyses

Correlation coefficients were calculated for the associations between the VO_2peak_ and plasma metabolites, with adjustments for known confounders, i.e., age and menopausal status (*), and additionally for phenotypical and clinical variables (**). While a graphical overview of all sex-specific bivariate correlations is provided in File S2, computed values are provided in Table S2. The number of plasma metabolites with correlations to the VO_2peak_ that were statistically significantly different from zero are shown, along with their categorization to major metabolic pathways (Table 1). The classification of VO_2peak_-correlated plasma metabolites to both major and specific metabolic pathways is moreover visualized in pie charts (File S3).

Confounder-adjusted correlation analyses revealed that 125 metabolites in females and 112 metabolites in males showed correlations with the VO_2peak_ that were significantly different from zero. Overall, only a limited number of common correlations were observed between the sexes, and generally stronger correlations could be found in the females. In women, 79 plasma metabolites were positively correlated with the VO_2peak_, among them 21 acyl-alkyl-phosphatidylcholine (PC ae) species (C44:3, C34:3, C42:4, C42:3, C42:2, C34:2, C40:3, C36:2, C44:6, C42:5, C36:3, C44:4, C42:1, C44:5, C40:5, C38:2, C40:4, C32:1, C32:2, C30:0, C40:1), 11 diacyl-phosphatidylcholine (PC aa) species (C42:2, C34:2, C36:2, C40:2, C42:0, C40:3, C42:4, C42:1, C28:1, C42:5, C36:3), sphingomyelin (SM) C16:0, lyso-phosphatidylcholine (lysoPC) C18:2, two acylcarnitines (C14:2, C10:2), the long-chain fatty acid (LCFA) C24:0, citrate, glyceric acid, acetate, and two unknown analytes, all demonstrating an r ≥ 0.25. The majority of the 46 negatively correlated metabolites in females were unknown, except for two LCFAs (C16:1 9cis, C18:1 11cis) with an r ≤ −0.25. In males, 33 plasma metabolites showed positive correlations with the VO_2peak_, including three lysoPCs (C18:2, C18:1, C17:0) and three unknown analytes with an r ≥ 0.25. Similar to females, most of the 79 negatively correlated metabolites in males remained unknown. Only two SMs (C18:0, C18:1) and diacyl-PC C40:6 with an r ≤ −0.25 could be identified. Overall, PCs largely showed weak to moderate positive correlations in females, whereas most PCs in males were either not or slightly negatively linked to the VO_2peak_. Significant bivariate correlations with the same directions in both sexes were observed for lysoPC C18:2 (F: r = 0.30; M: r = 0.34), glyceric acid (F: r = 0.29; M: r = 0.21), acetate (F: r = 0.26; M: r = 0.19), succinic acid (F: r = 0.24; M: 0.20), malic acid (r = 0.21; M: r = 0.22), and the LCFA C16:1 9cis (F: r = −0.32; M: r = −0.20), in addition to several unknown analytes with mainly negative correlations to the VO_2peak_ in both women and men (Table S2; File S2).

After additionally adjusting for phenotypical and clinical variables, 59 metabolites (0.20 ≤ |r| ≤ 0.39) in females and 24 metabolites (0.16 ≤ |r| ≤ 0.25) in males still exhibited weak to moderate correlations with the VO_2peak_ that were significantly different from zero. The majority of the VO_2peak_-related plasma metabolites in both females and males belonged to lipid metabolism, followed by AA metabolism and xenobiotics and related metabolism. However, only a few correlations with the same directions in both sexes were detected (e.g., U1.156). The top 10 of sex-specific positive and negative partial correlations between the VO_2peak_ and plasma metabolites are summarized in Table 2.

In females, the top 10 positively correlated plasma metabolites included five acyl-alkyl-PCs (C40:3, C42:4, C38:3, C36:2, C44:3), acylcarnitine C5, choline, glyceric acid, and acetylornithine, while the top 10 negatively correlated plasma metabolites were unknown analytes. In males, only seven plasma metabolites were positively correlated with the VO_2peak_, namely two AAs (alanine, glutamate), acylcarnitine C6 (C4:1-DC), diacyl-PC C36:3, and three unknown analytes. The top 10 negatively correlated plasma metabolites in males comprised eight unknown analytes, the xenobiotic tartaric acid and acyl-alkyl-PC C38:6. Sex-related differences in the direction of the relations were obvious for tartaric acid (F: r = 0.23; M: r = −0.21), diacyl-PC C42:1 (F: r = 0.23; M: r = −0.18), U2.250 (F: r = 0.11; M: r = −0.25), C5-carnitine (F: r = 0.31; M: r = −0.05), and alanine (F: r = −0.19; M: r = 0.18). While several PCs still showed weak to moderate positive correlations in females, single PCs in males tended to be slightly negatively linked to the VO_2peak_ (Table S2; File S2).

#### 2.4.2. Multivariate Association Analyses

The multivariate association between the VO_2peak_ and all 427 plasma analytes was assessed based on rank products obtained in cross-validated PLS models. Rank products were calculated by the geometric mean of the ranks of the regression coefficients of each metabolite in PLS models, across 20 random splits. For each metabolite variable, the importance of its contribution to the multivariate association was determined by permutation tests (see Section 4.7 (ii) for more details). A graphical overview of all sex-specific associations is provided in File S2. In Table S2, metabolites are listed together with their rank products of contribution to multivariate associations. Regarding females, PLS analysis showed that metabolites highly contributing to the confounder-adjusted multivariate association with CRF included several diacyl- and acyl-alkyl-PCs, the LCFA C16:1 9cis, as well as a number of unknowns. In males, metabolites with high contributions to the multivariate association comprised specific lysoPCs (C18:2, C18:1, C17:0), SMs (C18:0, C18:1), and diacyl-PC C40:6 next to unidentified analytes, when adjusting for age. As opposed to confounder-adjusted multivariate association analyses, the mean of the root mean square errors (RMSEs) based on the test samples was not always higher in the permutations than by using the original data, if additionally controlling for phenotypical and clinical variables (File S4). Consequently, the multivariate relationship between CRF and all 427 plasma analytes lost importance after applying additional adjustments.

**Figure 3:**
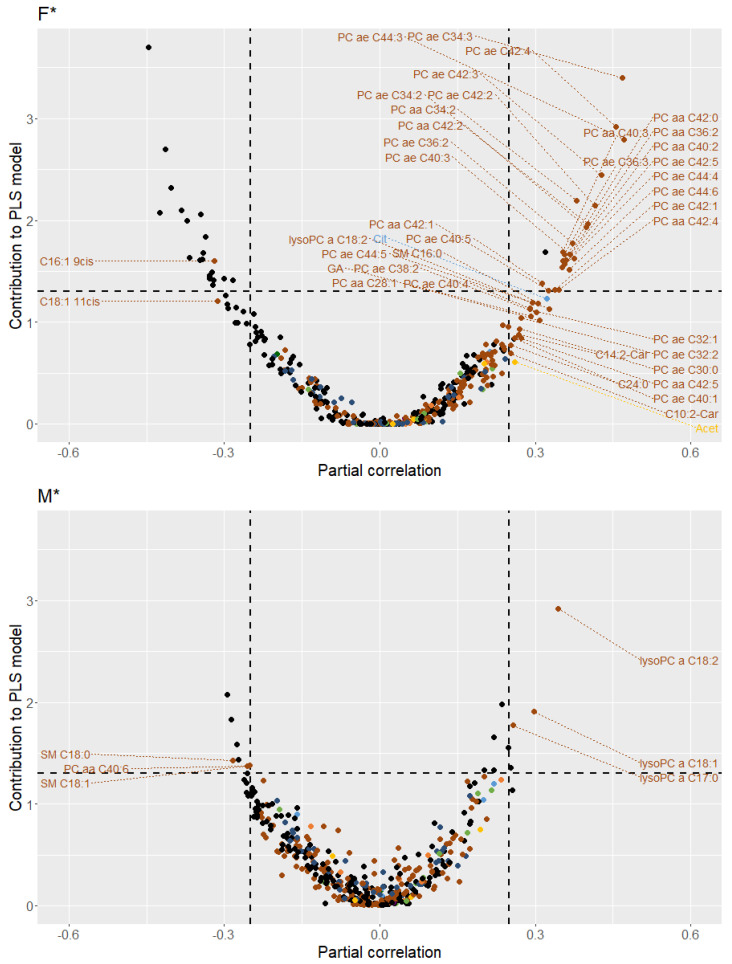
Volcano plots illustrating sex-specific plasma metabolite patterns associated with the VO_2peak_. F: females; M: males. * confounder (age/menopausal status)-adjusted. The y-axis represents the significance of the contribution of each metabolite variable to the multivariate association with the VO_2peak_, expressed as the negative logarithm of the relative frequencies of permutation-obtained rank products below measured rank products. The x-axis illustrates the direction and strength of partial correlations between the VO_2peak_ and metabolite variables, expressed as Pearson correlation coefficients (r) of Van der Waerden (VdW)-transformed variables. The classification of plasma analytes to major metabolic pathways is color-coded as follows: amino acid metabolism (dark blue); carbohydrate metabolism (yellow); cofactors and vitamins metabolism (dark green); energy metabolism (light blue); lipid metabolism (brown); mammalian-microbial cometabolism (orange); nucleotide metabolism (purple); xenobiotics and related metabolism (light green); unknown (black).

**Figure 4:**
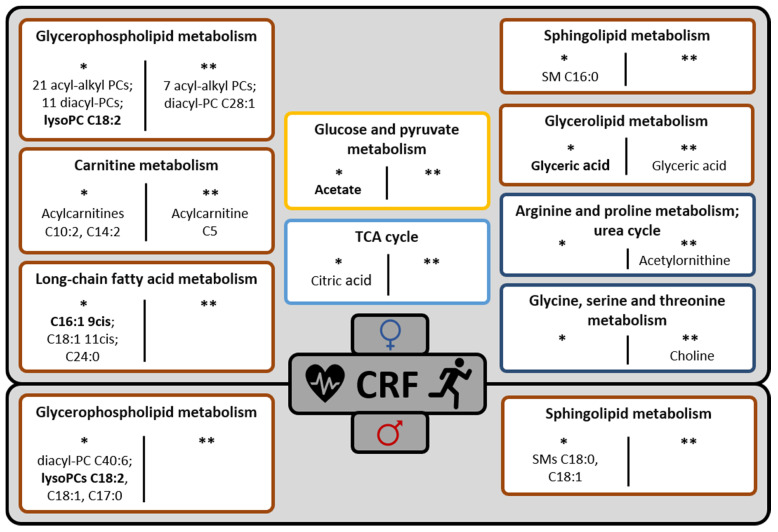
Classification of relevant CRF-associated plasma metabolites to major and specific metabolic pathways. Top: females; bottom: males. Major metabolic pathways are color-coded as follows: amino acid metabolism (dark blue); carbohydrate metabolism (yellow); energy metabolism (light blue); lipid metabolism (brown). * findings from confounder (age/menopausal status)-adjusted bi-/multivariate association analyses; ** findings from bivariate association analyses additionally adjusted for phenotypical/clinical variables. CRF: cardiorespiratory fitness; PC: phosphatidylcholine; SM: sphingomyelin. Metabolites with bivariate correlations to CRF that were significantly different from zero in both females and males are indicated in bold.

To visualize the sex-specific association patterns of plasma metabolites, the results of both bi- and multivariate association analyses were combined in a volcano plot (see Figure 3 for confounder-adjusted findings (*) and File S5 for confounder- and phenotypical/clinical variables-adjusted findings (**)). In the upper right and left corners, metabolites with moderate (|r| ≥ 0.25) bivariate correlations and significant contributions to multivariate associations are detectable. Metabolites in the upper middle region showed weak (|r| ≤ 0.25) bivariate correlations but significant contributions to multivariate associations, i.e., their relationship with the VO_2peak_ depended on all other considered analytes. In contrast, the lower right and left corners include metabolites with moderate bivariate correlations but no relevant contributions to multivariate associations, i.e., their relationship with the VO_2peak_ lost relevance if other metabolite variables with possibly redundant information were taken into account. For a subsequent metabolic interpretation of CRF-related metabolite patterns, plasma metabolites with either relevant bivariate correlations (|r| ≥ 0.25) or significant contributions to multivariate associations were considered (Figure 4).

#### 2.4.3. Multiple Regression Analyses

To additionally investigate the sex-specific relationship between the VO_2peak_ and selected metabolite variables in the presence of phenotypical and clinical data, multiple linear regression analyses were performed. By adjusting all included variables for age and menopausal status, the examined associations were independent of these non-modifiable variables. As described in the methods section, sex-specific models were calculated based on three different sets of phenotypical, clinical, and metabolite variables. The results of the cross-validated stepwise regression analyses are presented in Table S3.

Finally, the most suitable combination of phenotypical, clinical, and metabolite variables for a preferably good explanation of the VO_2peak_ was selected. The included variables (“approach 1–3 selection”) and the adjusted coefficients of determination (R^2^ (adjusted)) for the evaluation of the sex-specific final models are summarized in Table 3. With regard to approach 1 selection, seven phenotypical or clinical variables were selected for the final model of females, resulting in an R² (adjusted) of 0.40. For the final model of males, six phenotypical or clinical variables were selected, leading to an R^2^ (adjusted) of 0.43. When including all 21 phenotypical and clinical variables, an R^2^ (adjusted) of 0.36 for women and an R^2^ (adjusted) of 0.39 for men was obtained (see Table S3). With approach 2, the added value of plasma metabolites for explaining the confounder-adjusted VO_2peak_ in the presence of all 21 phenotypical and clinical variables was considered.

Nine or ten metabolites, respectively, were additionally selected for the final models, leading to a comparatively higher performance in both sexes (F: R^2^ (adjusted) = 0.72; M: R^2^ (adjusted) = 0.62). Contrary to approach 2, both phenotypical and clinical parameters, as well as metabolite variables, entered the model in a competing stepwise manner in approach 3. Actually, FM (%) was the only phenotypical/clinical variable being included in the final models of both sexes. In females, nine plasma analytes were additionally selected, resulting in an R^2^ (adjusted) of 0.68. In males, eight plasma analytes completed the model which finally showed an R^2^ (adjusted) of 0.59. In summary, approach 2, as well as approach 3, selection demonstrated an improved performance in both sexes, compared to the initial models solely based on phenotypical/clinical variables. While the acyl-alkyl-PC C40:3 was present in the females’ final models for both approaches 2 and 3, diacyl-PC C36:3, malic acid, as well as glutamate, were selected for the final models of males.

## 3. Discussion

The major finding of our systematic association analyses is that the VO_2peak_ was related to sex-specific sets of plasma metabolites that primarily belong to lipid metabolism. However, the observed correlations were rather moderate, and, independently of other clinical or phenotypical variables considered, only a small number of metabolites were significantly correlated with CRF. Multiple regression analyses revealed that models explaining the sex-specific VO_2peak_ could be improved when including selected plasma metabolites in addition to clinical and phenotypical parameters. For a metabolic interpretation of CRF-related metabolite patterns, a graphical overview of identified metabolites with relevant bivariate correlations (|r| ≥ 0.25) or significant contributions to multivariate associations with CRF and their pathway classification is provided in Figure 4.

Apart from detecting bi- and multivariate associations between CRF and plasma metabolites, which are discussed in the following sub-sections, we were also able to prove well-known relationships between CRF and several health-related clinical or phenotypical variables [4] in the KarMeN population. After correcting for age and menopausal status, we confirmed previous studies showing that CRF correlated negatively with the FM (%) [29], VATM [30], PWV [31], and TGs [32,33] and positively with HDL cholesterol [32,33] in both females and males. Equally consistent with the literature, but only present in men, were negative correlations of the age-adjusted VO_2peak_ with the HR_rest_ [5], diastolic BP [29,32], LDL cholesterol [34], and insulin [5]. In summary, our data provide evidence that even in a study sample consisting of metabolically healthy individuals and independent of age, individuals with higher CRF generally demonstrated lower values of clinical parameters, some of which are recognized as traditional risk factors for chronic metabolic or cardiovascular diseases.

### 3.1. Sex-Specific Plasma Metabolite Patterns Related to CRF

#### 3.1.1. Age- and Menopausal Status-Adjusted Findings

As shown by the results of age- and menopausal status-adjusted (*) correlation and PLS regression analyses, the CRF-related plasma metabolite pattern in females mainly comprised PCs, all of which were individually positively correlated with the VO_2peak_. PCs represent the most abundant phospholipid component in cellular membranes and plasma lipoprotein classes, being important for cell integrity or the assembly and stability of lipoproteins [35]. Besides, acyl-alkyl-PCs were proposed as antioxidants preventing lipoprotein oxidation [36]. Previous studies have reported lower levels of acyl-alkyl-PCs in obese or insulin-resistant individuals [36,37] and showed that specific acyl-alkyl-PCs were related to higher HDL cholesterol and a lower risk for type 2 diabetes [38]. In line with our results, Wientzek et al. demonstrated age- and sex-adjusted positive associations between CRF and several serum PCs in middle-aged adults [20]. Likewise, higher plasma levels of four acyl-alkyl-PCs were observed in adults with high CRF compared to less fit adults, when controlling for age and BMI [21]. While those relationships seemed to be independent of sex, associations between CRF and specific PCs in the KarMeN population were more pronounced in women than in men.

With regard to men, age-adjusted association analyses revealed that the CRF-related plasma metabolite pattern was dominated by three lysoPCs (C18:2, C18:1, C17:0), all of which were positively linked to the VO_2peak_, and two SMs (C18:0, C18:1), which were negatively linked to the VO_2peak_. In fact, lysoPC C18:2 also showed a relevant positive correlation with CRF in females. LysoPCs represent hydrolysis products from PCs, with relevant roles for cell signaling. As a major component of oxidized LDL, lysoPCs are also supposed to regulate the pathophysiological processes underlying atherosclerosis [39]. It is noteworthy that saturated lysoPCs are assumed to exert pro-inflammatory effects, whereas polyunsaturated lysoPCs such as C18:2 do not seem to possess inflammatory properties [40]. Actually, circulating lysoPCs were found to be reduced in obese individuals [41], and in particular, lysoPC C18:2 has been linked to a lower risk for type 2 diabetes [38] or cardiovascular disease [42]. As is consistent with our findings, Wientzek et al. showed positive correlations between CRF and serum lysoPCs C18:2 and C18:1, after controlling for sex and age. Thus, it is possible that lysoPC C18:2, in particular, might provide a potential link between CRF and its protective effects on chronic diseases. Our data moreover suggest a sex-dependent regulation of phospholipid metabolism in relation to CRF status. Although PC hydrolysis and lysoPC formation have been shown to be generally higher in men than in women (possibly due to differences in enzymatic activities, body composition, and/or hormonal or lifestyle factors [43]), the exact mechanisms underlying sex-related differences in CRF-associated phospholipids are largely speculative. However, we assume sex to be an important factor when studying how PC metabolism is linked to the health-beneficial effects of high CRF. In addition to lysoPCs, SMs are present in cell membranes or linked to lipoproteins [44]. Higher plasma SMs have been proposed as independent risk factors for cardiovascular diseases [45]. In particular, SMs with saturated acyl chains (C18:0 to C24:0) were closely correlated with parameters of obesity or insulin resistance [46]. As is consistent with our results, previous studies reported negative correlations between CRF and blood SM C18:0 [22,47] or SM C18:1 [20,47] in young [22] and middle-aged [20] adults or patients with coronary artery disease [47]. Despite sex-related differences, our findings indicate that even in healthy individuals and across a broad age range, higher CRF tends to be associated with lower values of potential novel blood biomarkers of the pathophysiological processes underlying cardiometabolic diseases.

Further relevant CRF-related plasma metabolites in females were two acylcarnitines (C14:2, C10:2), the LCFA C24:0, glyceric acid, acetate, and citric acid, all of which were positively linked to the VO_2peak_, and two LCFAs (C16:1 9cis, C18:1 11cis), which showed negative correlations with the VO_2peak_. Of those metabolites, palmitoleic acid C16:1 9cis also significantly contributed to the multivariate association with CRF. C16:1 9cis is an abundant fatty acid in human blood and adipose tissue. It can be ingested through diet or endogenously produced, and is assumed to act as a beneficial lipokine that prevents the negative effects of adiposity on insulin sensitivity [48]. Since circulating C16:1 9cis has been shown to be proportional to FM [48], the negative correlation between CRF and C16:1 9cis might be explained by the generally lower FM (%) in fitter females. Acylcarnitines are intermediates in the transport of LCFAs to mitochondria or byproducts of β-oxidation [49]. Although blood acylcarnitines have been identified as markers of insulin resistance, mitochondrial overload, and incomplete fat oxidation [50], they are also physiologically elevated in conditions with high lipolytic rates [51]. Recently, it has been shown that a training-induced rise in fasting levels of muscular long- and medium-chain acylcarnitines (e.g., C14:2 and C10:2) were related to improved CRF and potentially reflective of a more robust carnitine buffering system [52]. Glyceric acid is a sugar acid that connects several pathways, e.g., glycerolipid metabolism and glycolysis/gluconeogenesis. Even if its biological relevance with regard to PE has, to our knowledge, not yet been described, Lustgarten et al. also demonstrated a positive association between CRF and circulating glyceric acid in healthy females [22]. Equally in line with our results, blood acetate was shown to be higher in physically active adults [14]. Acetate is either directly formed from pyruvate, providing a source for acetyl-coenzyme A [53], or can be produced by the intestinal microbiota, finally entering circulation [54]. It has been suggested that a higher PF is linked to a greater abundance of gut bacteria with positive health effects, being reflected in the release of fermentation metabolites like acetate [55,56]. In addition to its anti-inflammatory and vasodilatory properties [54], acetate has been proposed as an important energy substrate during endurance exercise in mice [57]. However, the extent to which the microbiome indeed affects the PE capacity in humans, and whether resting blood acetate might mirror this association, requires further investigation. While the tricarboxylic acid (TCA) cycle intermediate citric acid only showed a relevant positive correlation with the VO_2peak_ in females, malic acid and succinic acid tended to be positively linked to CRF in both sexes. Previous studies revealed a rise in fasting plasma malic acid after weight loss and PE intervention in obese women [58], a training-induced increase in muscular succinic acid in subjects at risk of metabolic disease [52], or a slightly positive correlation between CRF and serum succinic acid in healthy young men [59]. Despite weak bivariate correlations, our results support the suggestion that specific TCA cycle intermediates might be potentially interesting blood markers of the beneficial effects of chronic PA occurring at a muscular level, such as an increased mitochondrial density or TCA cycle capacity.

To conclude, we provided evidence of sex-specific CRF-associated plasma metabolite patterns after adjusting for age and menopausal status. Sex-related differences were especially observed for lipid metabolism-related PCs, which generally showed relevant associations with the VO_2peak_ in females but not in males. However, weak to moderate correlations between CRF and lysoPC C18:2, LCFA C16:1 9cis, glyceric acid, as well as the energy or carbohydrate metabolism-related succinic acid, malic acid, or acetate, were observed in both sexes. Accordingly, our results suggest that CRF-related adaptations in glycerophospholipid metabolism might vary between sexes, whereas the consequences of a high CRF on, e.g., the TCA cycle seem to be present in females and males.

#### 3.1.2. Age-, Menopausal Status- and Phenotypical/Clinical Variables-Adjusted Findings

A higher CRF is generally concomitant with a healthier body composition, adaptations in heart and arterial function, or cardiometabolic risk parameters. Hence, we next aimed to identify metabolite patterns that were independent of further assessed phenotypical and clinical variables associated with CRF. When adjusting for potential covariates (**), a comparatively smaller number of metabolites were correlated with the VO_2peak_ in both sexes. Thus, it can be suggested that many of the observed associations cannot be primarily and specifically attributed to CRF. As no relevant CRF-related information seemed to remain in the overall plasma metabolite profile, findings from PLS regression analyses are not further discussed.

After applying additional adjustments, seven acyl-alkyl-PCs, C5-carnitine, choline, glyceric acid, acetylornithine, and diacyl PC C28:1 still showed moderate positive correlations with the VO_2peak_ in females. Two of these acyl-alkyl-PCs (C40:3, C44:3) were also present in a cluster of 19 PCs that were related to CRF in the study of Wientzek et al. when controlling for sex, age, BMI, and waist circumference, among others [20]. The fact that we adjusted for more precise body composition measures and parameters of lipoprotein metabolism, which are known to be linked to both CRF [60] and PCs [35,38], could explain the study-specific results. Choline participates in multiple pathways of lipid or AA metabolism, serving as a precursor for PC species, the neurotransmitter acetylcholine, or betaine. Circulating choline can result from diet or PC breakdown [61] and was shown to be either lower [18] or higher [59,62] in fitter individuals. Even though a more efficient conversion of PCs to choline in trained individuals was assumed [62], it cannot be excluded that female KarMeN subjects with high CRF were also characterized by a higher dietary choline intake. In summary, sex-specific differences in CRF-related plasma metabolites were still detectable, e.g., for some PCs that remained positively correlated with female CRF but were not or even slightly negatively linked to male CRF. Moreover, the xenobiotic tartaric acid showed sex-specific correlations with CRF. This might be due to differences in the dietary intake of wine, vinegar, or grapes [63,64] in more or less fit women or men. The AA alanine showed a slightly negative correlation to the female VO_2peak_ but a slightly positive correlation to the male VO_2peak_. In the literature, circulating alanine was mostly negatively linked to CRF status [15,23].

### 3.2. Sex-Specific CRF Explanation Models

Contrary to a previous attempt to explain the variability of CRF in the KarMeN population based on urinary metabolites [65], we were now able to identify sets of plasma metabolites that, together with clinical and phenotypical variables, contributed to a relatively good explanation of the VO_2peak_ in both sexes, after adjusting for age and menopausal status. In all approaches, the FM (%) entered the models as the first variable, already explaining 33.5% (females) or 42.3% (males) of the confounder-adjusted VO_2peak_. Six (females) or five (males) further phenotypical or clinical parameters slightly improved the models (approach 1 selection). However, when phenotypical and clinical parameters, as well as metabolite variables, entered the models in a competing manner, plasma analytes led to fairly improved performance in both sexes, as demonstrated by an R^2^ (adjusted) of 0.68 for females and 0.59 for males (approach 3 selection). Regarding females, acyl-alkyl-PC C40:3 was the second most important determinant of the VO_2peak_. Interestingly, this PC sum parameter also showed relevant bi- and multivariate associations with CRF. As the relationship between acyl-alkyl-PC C40:3 and the VO_2peak_ did not appear to be influenced by other assessed plasma analytes, and the bivariate correlation persisted independently of adjustments, the ability of acyl-alkyl-PC C40:3 to predict CRF in females should be given special consideration in further studies. Regarding males, the TCA cycle intermediate malic acid was the second variable being included in the model after the FM (%) (approach 3 selection). In a study by Lustgarten et al., blood metabolites-based CRF explanation models were also sex-dependent. Seven serum metabolites in females and five serum metabolites in males explained 58 or 80% of CRF variability, respectively. However, as regression models were not adjusted for age, body composition, diet, or PA [22], it is uncertain if those metabolites are specifically indicative of CRF. As demonstrated by our stepwise regression models, the VO_2peak_ was largely determined by the FM (%) in the KarMeN study, supporting the assumption that some associations between CRF and plasma metabolites might be mechanistically linked to clinical or phenotypical traits that were also influenced by chronic PA. In fact, Kujala et al. have shown that comparatively few blood metabolites remained significantly associated with CRF after adjusting for the body fat content in healthy young men [17]. Nevertheless, our findings could emphasize the additional value of metabolomics data for explaining the variability inherent in the VO_2peak_, hinting at some possibly relevant plasma metabolites that could help to infer an individual’s CRF status. Further studies will be needed to verify if those metabolites are indeed specific for CRF, and to what extent they are influenced by other functional or morphological characteristics of the human organism.

### 3.3. Strengths and Limitations

The major strength of this study is that it provides a systematic overview of the relationship between CRF and the plasma metabolome in a relatively large population consisting of both women and men, with a wide age range. The KarMeN study was characterized by highly standardized anthropometric and clinical examinations, as well as a strictly controlled procedure for blood collection, CRF assessment, and metabolomics analyses. To minimize the variability in metabolomics measurements, plasma samples were collected in the fasting state, and pre-menopausal women were examined within the luteal phase of their menstrual cycle. As the KarMeN study focused on healthy, non-smoking subjects with a normal to moderately high weight, excluding individuals with supplement use or hormonal treatment, the metabolic variation related to diseases, medication, or metabolic disorders was also markedly reduced. To control for known confounders from the very beginning, we conducted sex-specific analyses adjusted for age and menopausal status. Owing to the comprehensive characterization of study participants, further potential confounding factors related to body composition, clinical blood biochemistry, lung and arterial function, short-term and habitual PA, as well as diet could be considered. Another strength of this study is the applied multi-platform metabolomics approach, allowing the detection of a large number of plasma analytes from a broad range of biochemical classes and pathways. Limitations of the study include the cross-sectional design, as it does not allow the deriving of causal relationships. Furthermore, some of the plasma analytes showing relevant associations with CRF could not be identified with sufficient certainty and thus, regrettably, remained unknown.

## 4. Materials and Methods

### 4.1. Subjects and Study Design

The cross-sectional KarMeN study was conducted between March 2012 and July 2013 at the Division of Human Studies of the Max Rubner-Institut in Karlsruhe, Germany. Details on inclusion and exclusion criteria, as well as a comprehensive description of the study design and examination procedures, have already been published [26]. Briefly, 301 healthy, non-smoking individuals (172 men, 129 women) between 18 and 80 years of age were included. All subjects visited the study center three times and were thoroughly characterized by anthropometric, clinical, and functional examinations. Moreover, data on PA, diet and the menopausal status of female subjects were collected. Since the menstrual cycle is known to affect metabolite profiles [66], all premenopausal women were scheduled for examinations within their luteal phase. On the morning of the second study day, fasting plasma samples were collected using 9 mL EDTA plasma tubes (S-Monovette, Sarstedt, Nümbrecht, Germany). The plasma samples were immediately centrifuged at 1850× *g* at 4 °C, aliquoted into small portions, and cryopreserved at −196 °C until metabolomics analyses. Serum samples (S-Monovette Z-gel, Sarstedt, Nümbrecht, Germany) were collected for standard clinical biochemistry analyses.

### 4.2. Anthropometry and Body Composition Assessment

Bodyweight and height were measured in underwear and without shoes (Seca 285, Hamburg, Germany), and the BMI was calculated by dividing the body weight in kilograms by the height in meters squared. The body composition was assessed by dual-energy X-ray absorptiometry (Lunar iDXA, GE Healthcare, München, Germany) and the LBM, FM, and VATM, as well as the bone mineral content (BMC), were determined with the supplementary software enCOREv16. The FM (%) was calculated by dividing the total FM by the total body weight. Approval for dual-energy X-ray absorptiometry measurements was received from the Federal Office for Radiation Protection (Z5-22462/2-2011-043).

### 4.3. PF and PA Assessment

As a measure of PF, CRF was assessed by a standardized incremental exercise test on a bicycle ergometer (Ergobike medical, Daum, Fürth, Germany). All participants started pedaling at 25 Watts and the workload was then augmented by 25 Watt every 2 min until individual exhaustion, as previously described [65,67]. The respiratory gas exchange was measured breath-by-breath by using a cardiopulmonary exercise testing system (MetaMax 3B, Cortex, Leipzig, Germany). Since the VO_2max_ could not be determined with certainty, as it requires the presence of a plateau in oxygen uptake, the VO_2peak,_ as the highest attained oxygen uptake during the test, was assessed and expressed relative to the bodyweight in mL kg^−1^ min^−1^. During the entire procedure, the heart rate was recorded (T31 coded, Polar Electro GmbH Deutschland, Büttelborn, Germany). In addition, continuous hemodynamic monitoring was conducted by running a 12-channel electrocardiogram (CardioDirect 12, DelMar Reynolds GmbH, Feucht, Germany) and by measuring the BP every 2–3 min on the right upper arm (Boso-Carat Professional, Bosch + Sohn, Jungingen, Germany). As measures of PA, both short-term and habitual PA were determined [67]. Briefly, the level of short-term PA was assessed during a period of seven consecutive days by combined accelerometry and heart rate measurements (Actiheart, CamNtech, Cambridge, UK). The average AEE during the study week was finally calculated by the supplied software (Version 4.0.103) and given in kcal/day. To obtain the habitual PA for the last three months, participants filled out the standardized international physical activity questionnaire. The average weekly PA was calculated and expressed in MET-min/week.

### 4.4. Dietary Assessment

Food consumption for the day prior to blood sampling was assessed by conducting a 24-h recall in a personal interview, using the software EPIC-Soft, as described in detail elsewhere [26,68]. In order to evaluate the diet quality of participants, a modified version of the Healthy Eating Index was calculated, which was initially applied in the second German National Nutrition Survey (“Nationale Verzehrsstudie (NVS) II”) [69] and adapted with minor modifications in the KarMeN study. The so-called HEI-NVS evaluates the overall diet quality, with scores ranging from 0 (low quality) to 110 (high quality).

### 4.5. Clinical Examinations

Clinical parameters like the HR_rest_, as well as systolic and diastolic BP, were measured after a resting period of at least five minutes in a sitting position (Boso-Carat Professional, Bosch & Sohn, Jungingen, Germany). The pulmonary function was assessed by spirometry (FlowScreen, CareFusion, Hoechberg, Germany) and the VC_max_, as well as the FEV1, were recorded. Moreover, arterial stiffness was determined (ARTERIOGraph, Medexpert, Budapest, Hungary) and the PWV was calculated. Standard clinical biochemistry analyses in fasting serum samples (e.g., hemoglobin (Hb), glucose, HbA1c, TGs, HDL- and LDL-cholesterol) were carried out by the certified medical laboratory MVZ Labor PD Dr. Volkmann (Karlsruhe, Germany) and insulin concentrations were determined with an enzyme-linked immunosorbent assay (ME E-0900, LDN, Nordhorn, Germany).

### 4.6. Metabolomics Analyses

To obtain a broad coverage of the plasma metabolome, a number of complementary (non-)targeted analytical techniques were applied. Quality control (QC) samples prepared by pooling plasma samples from all KarMeN participants were analyzed, along with plasma samples in all applied methods. The following section provides a brief summary of the different methods; further details are available in the supplement of Rist et al. [28].

**Non-targetedtwo-dimensional gas chromatography (GC × GC)-MS analysis.** Plasma samples were analyzed by a non-targeted GC × GC-MS-based approach using a Shimadzu GCMS QP2010 Ultra instrument equipped with a ZOEX ZX2 modulator [70]. The GC × GC-MS raw data files were then processed by non-targeted alignment using in-house developed R-modules [71]. By means of regularly injected QC samples, signal intensity drift, i.e., intra- and interbatch effects occurring during the measurement period, were corrected. With this method, a wide range of metabolites (including AAs, amines, organic acids, sugars, sugar alcohols, or polyols) could be detected.

**Targetedgas chromatography (GC)-MS analysis of fatty acids.** The chromatographic separation of plasma fatty acids usually requires the application of specialized polar columns and can thus not be conducted adequately by using a standard apolar × medium-polar GC × GC column setup. Therefore, a previously described method [72] was applied to detect plasma fatty acids such as methyl esters, with minor modifications. By using a GC single quadrupole instrument (Shimadzu GCMS QP2010 Ultra) and a BPX90 column (Trajan Scientific), 48 fatty acids were finally determined in a quantitative manner.

**Liquid chromatography (LC)-MS metabolite profiling using the Absolute IDQ™ p180 kit.** Plasma samples were also utilized for targeted metabolite profiling using the Absolute IDQ™ p180 kit developed by Biocrates AG (Innsbruck, Austria). The general preparation and quantification procedure has already been described [73]. For the chromatographic separation of AA and biogenic amines, a Zorbax Eclipse XDB-C18 column (3 × 100 mm, 3.5 μm; Agilent, Waldbronn, Germany) equipped with a SecurityGuard™ column (C18, 4.0 × 3.0 mm; Phenomenex, Aschaffenburg, Germany) was used. PCs and SMs were analyzed by flow injection analysis into the analytical system, comprising a Nexera UHPLC system (Shimadzu) coupled to an API QTRAP15500 mass spectrometer (AB Sciex, Darmstadt, Germany). With this method, a variety of acylcarnitines, AAs, biogenic amines, SMs, and PCs were detected. While lysoPC species always have one acyl-bound fatty acid, other included PC species are characterized by two acyl-bounds (PC aa) or one acyl- and one alkyl-bound (PC ae) fatty acid, respectively. In general, each analyzed PC represents a sum parameter of different PC species with identical residue sums (e.g., PC ae C32:1 may consist of PC ae C16:0/C16:1, PC ae C18:0/C14:1, etc.).

**Targeted LC-MS analysis of methylated amino compounds.** The quantification of seven amino compounds in plasma was conducted by ultra-performance liquid chromatography-tandem MS, using an Acquity H-Class UPLC coupled to a Xevo TQD triple quadrupole MS (both from Waters, Eschborn, Germany), as previously established [74]. Plasma samples were diluted with acetonitrile after protein precipitation and separated by an inverse acetonitrile gradient on a polar hydrophilic interaction liquid chromatography column (Acquity BEH Amide, Waters, Eschborn, Germany). Target analytes, as well as deuterated internal standards, were monitored by positive electrospray ionization in multiple reaction monitoring mode.

**Targeted LC-MS analysis of bile acids.** 14 bile acids in plasma samples were quantified using a 1200 series HPLC system (Agilent, Waldbronn, Germany) coupled to a Q-Trap 3200 MS (AB Sciex, Darmstadt, Germany) [75].

**Non-targeted NMR analysis.** Plasma samples were analyzed by 1D-^1^H-NMR spectroscopy. Briefly, they were measured at 310 K on an AVANCE II 600 MHz NMR spectrometer equipped with a 1H-BBI probe head and a BACS sample changer (Bruker BioSpin GmbH, Rheinstetten, Germany). All obtained plasma spectra were automatically phased with the Bruker AU program apk0.noe. Using the program AMIX 3.9.14 (Bruker BioSpin GmbH, Rheinstetten, Germany), they were then referenced to the EDTA signal and bucketed graphically, so that buckets contained only one signal or group of signals and no peaks were split between buckets, whenever possible. Buckets were either annotated to a previously known and identified analyte, or registered as unknown. The identification of metabolites was carried out with Chenomx NMR Suite 8.1 (Chenomx, Edmonton, Canada). The detected analytes included organic acids, AAs, amines, and sugar alcohols.

### 4.7. Data Handling and Statistical Analysis

The data from the different analytical platforms were integrated into a common data matrix, consisting of 301 samples and 657 plasma analytes. With respect to the study participants, we excluded 49 individuals due to missing spiroergometry data (n = 40), technical errors during analyses (n = 7), implausibly low HR_rest_ data (n = 1), and a missing plasma sample (n = 1). If identified metabolites were measured by more than one of the analytical platforms, those metabolites that were detected by the less quantitative method were excluded (n = 149). Further analytes were deleted if they had a detected frequency lower than 20% in either the female or male subgroup (n = 81). Thus, the final data matrix contained 252 individuals and 427 plasma analytes. The dataset, including metabolite data and metadata, is provided in Table S1. Prior to statistical analyses, metabolite data were transformed into Van der Waerden (VdW) scores. By using this rank-based inverse normal transformation, the data were converted into ranks, transformed to a scale between 0 and 1, and then, the corresponding standard normal quantiles were calculated. This transformation took the issue of values below the limit of detection into account and led to a uniform scale for all analytes, i.e., they were finally comparable between analytical platforms.

Based on sex-specific VO_2peak_ quartiles, the sex-specific VO_2peak_ data were divided into four quarters (q), and differences in basic characteristics between the subgroups of the corresponding participants of the quarters were examined by Welch ANOVA (chi-squared test) for numeric (categorical) variables. All subsequent statistical analyses were conducted separately for sexes and the non-modifiable factors age (and menopausal status in females) were treated as confounders. Random forest regression algorithms considering age, phenotypical, and clinical variables were applied to impute missing values for AEE and PWV. Similar to the metabolite data, both the VO_2peak_ and considered phenotypical and clinical parameters were transformed into VdW scores prior to statistical analyses. To examine the sex-specific relationship between the VO_2peak_ and selected phenotypical and clinical parameters, Pearson correlations adjusted for age (and menopausal status in females) were calculated. Correlations were considered statistically significantly different from zero when the 95% confidence intervals (CIs) did not include zero.

Regarding metabolomics data analysis in sex-specific subgroups, three major aims were pursued: (i) to investigate the relationship between the VO_2peak_ and single plasma metabolites (bivariate association analyses), (ii) to determine the relationship between the VO_2peak_ and all plasma metabolites simultaneously (multivariate association analyses) and (iii) to identify a set of plasma metabolites possibly improving the explanation of the VO_2peak_ in the presence of phenotypical and clinical data (multiple regression analyses).

**(i)**  **Bivariate association analyses**

To examine the sex-specific relationship between the VO_2peak_ and single plasma metabolites, adjusted for age and menopausal status, partial Pearson correlation coefficients (r) with 95% CIs were calculated. In a second step, correlations independent of further phenotypical and clinical variables were assessed. More specifically, we performed sex-specific correlation analyses by adjusting not only for the above-mentioned confounders but also for the following phenotypical and clinical parameters: LBM, FM (%), VATM, BMC, height, Hb, glucose, insulin, HbA1c, TGs, HDL and LDL cholesterol, HR_rest_, systolic and diastolic BP, PWV, VC_max_, FEV1, AEE, total MET, and HEI-NVS.

**(ii)**  **Multivariate association analyses**

To analyze the relationship between the VO_2peak_ and all 427 plasma metabolite variables simultaneously, PLS regression analyses using nested cross-validation were conducted separately for women and men. The PLS analyses were either applied on confounder-adjusted metabolite variables or on metabolite variables additionally adjusted for the above-listed phenotypical and clinical parameters. The outer loop contained 20 random splits in a calibration dataset (containing 80% of all samples) and a test dataset (containing the remaining 20% of all samples). The data were preprocessed, including the formation of VdW-scores, respective adjustments, and unit variance scaling based on the calibration data. As the inner loop, a single random eight-fold cross-validation was used to tune the PLS model, based on the RMSE. Thereby, the number of predictive components was restricted to being at most ten. A rank for the obtained PLS regression models was assigned to each metabolite variable according to the negative absolute value of its regression coefficient. By calculating the geometric means of the ranks across the 20 random splits, a final rank product for each metabolite variable was obtained. The model performance was evaluated by the mean of RMSEs on the test samples across the 20 random splits. Moreover, 2500 permutations of the VO_2peak_ values were run, and the relative frequency of permutation-obtained rank products below the previously calculated rank products was assessed. If the relative frequency was ≤0.05, the contribution of a metabolite variable to the VO_2peak_ in a multivariate association was considered significant.

**(iii)**  **Multiple linear regression analyses**

To assess the relationship between the VO_2peak_ and sets of phenotypical, clinical, and metabolite variables, three different exploratory multiple linear regression models were calculated for each sex, with the confounder-adjusted VO_2peak_ as the dependent variable, and with stepwise forward-selected confounder-adjusted clinical, phenotype, and metabolite variables as independent variables. In detail, the following approaches were applied:

- Approach 1: Only phenotypical/clinical variables (n = 21) were stepwise selected.
- Approach 2: All Phenotypical/clinical variables (n = 21) were included and only plasma metabolite variables (n = 427) were stepwise selected.
- Approach 3: Phenotypical/clinical variables (n = 21) as well as plasma metabolite variables (n = 427) were stepwise selected.

While in approach 2, all phenotypical/clinical variables entered the model as fixed variables before considering the plasma metabolites, in approach 3 all variables entered the model in a competing manner. To obtain a ranking of confounder-adjusted phenotypical, clinical, or metabolite variables according to their contribution for explaining the adjusted VO_2peak_, the models were built by maximizing the coefficients of determination (R^2^).

In addition to the ranking of variables, a single linear multiple regression model was calculated for each of the approaches 1 to 3 in order to obtain a manageable number of variables that, in combination, explained CRF. For variable selection, the previously described stepwise multiple linear regression analyses were performed on a calibration dataset (containing 80% of all samples) and the predictive accuracy of each step was assessed on the test dataset (containing the remaining 20% of the samples). The selection was stopped if the predictive accuracy decreased for the first time. In total, the analysis was repeated 1000 times with random assignments of samples into calibration and test datasets. Finally, the number of times each variable was present in those cross-validated stepwise regression models was counted (a relative frequency of 1 means that the variable was always considered in stepwise variable selection). All variables with a relative frequency ≥ 0.05 were then included in a final model with respect to approaches 1 to 3. The obtained final models were described by the adjusted R² and compared within each subpopulation. Statistical analysis was performed by using SAS JMP 11.0.0 (SAS Institute Inc. 2013, Cary, NC, USA) and the software R Version 4.0.0 [76], using the packages named caret [77], openxlsx [78], and missForest [79]. Figures were generated in Excel, 2016 or R, using the packages named ggplot2 [80], ggrepel [81], and ggpubr [82]. Supplementary PDF files were generated with LaTeX, using the package knitr [83]. R-scripts for the calculation of results and generation of figures and PDF files are provided in Files S6–S9.

### 4.8. Metabolite Classification

For the biological interpretation of CRF-related metabolite profiles, identified and putatively annotated metabolites, i.e., compounds with Metabolomics Standards Initiative (MSI)-level 1 or 2 [84], respectively, were manually assigned to 8 major and 32 specific pathways of human metabolism based on the information provided by the human metabolome database 4.0 [85] and the Kyoto Encyclopedia of Genes and Genomes PATHWAY database [86]. Annotation of analytes detected using untargeted approaches, showing relevant associations with CRF, was performed as follows: spectra of GC × GC-MS analytes were matched against an in-house spectral library as well as against the FiehnLib and the NIST17 libraries. If matching was unsuccessful, structural hypotheses were derived depending on the presence of known diagnostic fragments (see File S9 in Ulaszewska et al. [87]) and additionally, especially in the case of sugars and sugar-like compounds, based on the compound’s position in the two-dimensional chromatogram. Unfortunately, the spectra of CRF-related unknown analytes were mostly unspecific, which hampered structural elucidation. In the case of NMR, the identification of CRF-associated unknown analytes was not possible because the particular buckets contained either unspecific signals or overlapped peaks.

## 5. Conclusions

In summary, our findings demonstrated sex-dependent relationships between CRF and specific plasma metabolites in the KarMeN population. Apart from proving well-known associations between CRF and, further, partly health-related phenotypical or clinical variables in both sexes, we could identify a number of PCs, lysoPCs, and SMs as being associated with the VO_2peak_ in either females or males, when controlling for age and menopausal status. However, independently of selected clinical or phenotypical variables, sex-specific CRF tended to be correlated with a rather small number of plasma metabolites primarily related to lipid-, AA-, or xenobiotics-related metabolism. Hence, many of the observed associations between CRF and metabolites were likely to be mediated by the considered clinical or phenotypical parameters. Although the variability of CRF was largely determined by the FM (%) in both sexes, our stepwise regression analyses revealed certain sets of plasma metabolites able to improve sex-specific VO_2peak_ explanation models. In particular, acyl-alkyl-PC C40:3 could be identified as a possibly interesting metabolite parameter for conclusions on CRF status in healthy females. Remarkably, CRF-associated metabolites have already been discussed as being reflective of exercise-induced adaptations in muscular energy metabolism (e.g., malic acid, succinic acid, acylcarnitines) or inversely linked to the development of cardiometabolic diseases (e.g., PCs, lysoPCs). Therefore, those metabolites might represent potential mediators of the performance- or health-enhancing effects of chronic PA. However, more research is needed to further clarify the mechanisms and metabolic pathways underlying sex-specific differences in CRF-associated metabolite profiles. Finally, we recommend future studies examining blood metabolic markers related to CRF to conduct sex-separated analyses and to consider age, menopausal status, body composition, and other health-related variables as covariates.

## Figures and Tables

**Table 1:** The number of detected plasma metabolites, according to major metabolic pathways, and number of metabolites significantly correlating with the VO_2peak_, shown separately by sex.

Pathway	Total Number of Plasma Metabolites	Number of Plasma Metabolites Correlating with the VO_2peak_			
		Females (n = 102)		Males (n = 150)	
		*	**	*	**
**All**	427	125	59	112	24
Lipid metabolism	164	63	27	36	8
Amino acid metabolism	41	4	6	8	2
Xenobiotics and related metabolism	12	2	2	4	1
Mammalian-microbial cometabolism	7	2	0	1	0
Carbohydrate metabolism	5	2	1	1	0
Energy metabolism	5	3	1	2	0
Cofactors and vitamins metabolism	1	1	0	0	0
Nucleotide metabolism	1	0	0	0	0
Unknown	191	48	22	60	13

* confounder (age/menopausal status)-adjusted; ** additionally adjusted for 21 phenotypical/clinical variables.

**Table 2:** Top 10 of sex-specific partial correlations between the VO_2peak_ and plasma metabolites.

	Positive Correlations		Negative Correlations	
	Variables	r (95% CIs)	Variables	r (95% CIs)
Females				
1	PC ae C40:3	**0.37 (0.19; 0.53)**	U3.961	**−0.39 (−0.55; −0.22)**
2	PC ae C42:4	**0.31 (0.13; 0.48)**	U3.956	**−0.32 (−0.49; −0.14)**
3	C5-Carnitine	**0.31 (0.12; 0.47)**	U3.950	**−0.31 (−0.48; −0.12)**
4	PC ae C38:3	**0.28 (0.09; 0.45)**	U4.252	**−0.31 (−0.47; −0.12)**
5	Choline	**0.27 (0.08; 0.44)**	U3.971	**−0.31 (−0.47; −0.12)**
6	Glyceric Acid	**0.27 (0.08; 0.44)**	U0978	**−0.30 (−0.46; −0.11)**
7	U0856	**0.27 (0.08; 0.44)**	U0975	**−0.28 (−0.45; −0.09)**
8	PC ae C36:2	**0.26 (0.07; 0.44)**	U2.656	**−0.28 (−0.45; −0.09)**
9	Acetylornithine	**0.26 (0.07; 0.43)**	U1.156	**−0.28 (−0.45; −0.09)**
10	PC ae C44:3	**0.26 (0.07; 0.43)**	U3.060	**−0.26 (−0.43; −0.07)**
Males				
1	U0130	0.19 (0.03; 0.34)	U2.250	**−0.25 (−0.39; −0.09)**
2	Alanine	0.18 (0.02; 0.33)	U2.822	−0.21 (−0.36; −0.05)
3	C6 (C4:1-DC)-Carnitine	0.18 (0.02; 0.33)	U (Sugar-like 4)	−0.21 (−0.36; −0.05)
4	U2.910	0.18 (0.02; 0.33)	U1331	−0.21 (−0.36; −0.05)
5	PC aa C36:3	0.18 (0.02; 0.33)	Tartaric acid	−0.21 (−0.36; −0.05)
6	U3.385	0.17 (0.01; 0.32)	U1.156	−0.20 (−0.35; −0.04)
7	Glutamate	0.17 (0.01; 0.32)	U0.936	−0.19 (−0.34; −0.03)
8	-	-	U1.159	−0.19 (−0.34; −0.03)
9	-	-	U1.166	−0.19 (−0.34; −0.03)
10	-	-	PC ae C38:6	−0.18 (−0.33; −0.02)

Pearson correlations were performed on Van der Waerden (VdW)-transformed data and results of partial correlations adjusted for age, menopausal status, and further phenotypical/clinical variables are presented. Pearson correlation coefficients (r) and the lower and upper limit of the 95% confidence intervals (CIs) are rounded to two decimal places. |r| ≥ 0.25 are indicated in bold. PC aa: diacyl-phosphatidylcholine; PC ae: acyl-alkyl-phosphatidylcholine; U: unknown. For unknown NMR-analytes, the chemical shift of the lower bucket border is indicated in ppm.

**Table 3:** Summary of sex-specific final models for the confounder-adjusted VO_2peak_.

Model	Females (n = 102)	Males (n = 150)
Approach 1 Selection	R² (adjusted) = 0.40 FM (%), HDL cholesterol, LBM, PWV, Hb, BP systolic, BP diastolic	R² (adjusted) = 0.43 FM (%), HDL cholesterol, BMC, AEE, TGs, LDL cholesterol
Approach 2 Selection	R² (adjusted) = 0.72 All phenotypical/clinical variables + *PC ae C40:3, U3.961, S-Methylcysteine, Tartaric acid, U1.148, Serine, C24:0, Kynurenine, U0992*	R² (adjusted) = 0.62 All phenotypical/clinical variables + *PC aa C36:3, U0130, Tartaric acid, C6 (C4:1-DC)-Carnitine, C14:1-OH-Carnitine, U2.250, Malic acid, Glutamate, C24:0, U1.226*
Approach 3 Selection	R² (adjusted) = 0.68 FM (%), *PC ae C40:3, myo-Inositol, U0975, U3.961, U7.294, Glycine, U2.313, Lysine, C18:1-Carnitine*	R² (adjusted) = 0.59 FM (%), *Malic Acid, Taurocholate, PC aa C36:3, U0130, PC aa C36:6, Glutamate, U(Similar to Uracil), U1.226*

Variables were selected based on the results of the stepwise regression analyses and included in sex-specific final models. All variables were Van der Waerden (VdW)-transformed and adjusted for age (and menopausal status in females). Selected metabolite variables are indicated in italics. Approach 1: only phenotypical/clinical variables (n = 21) were stepwise selected; approach 2: all phenotypical/clinical variables (n = 21) were included and only plasma metabolite variables (n = 427) were stepwise selected; approach 3: phenotypical/clinical variables (n = 21) as well as plasma metabolite variables (n = 427) were stepwise selected. AEE: activity energy expenditure; BMC: bone mineral content; BP: blood pressure; FM: fat mass; Hb: hemoglobin; HDL: high-density lipoprotein; LBM: lean body mass; LDL: low-density lipoprotein; PC aa: diacyl-phosphatidylcholine; PC ae: acyl-alkyl-phosphatidylcholine; PWV: pulse wave velocity; R^2^ (adjusted): adjusted coefficient of determination; TGs: triglycerides; U: unknown.

## Data Availability

Data is contained within the article or Supplementary Material.

## Supplementary Materials

The following are available online at https://www.mdpi.com/article/10.3390/metabo11070463/s1, File S1: Basic characteristics of the KarMeN participants according to VO_2peak_ quarters, File S2: Graphical overview of associations between the VO_2peak_ and plasma metabolites, File S3: Classification of metabolites with significant bivariate correlations to metabolic pathways, File S4: Evaluation of PLS approaches, File S5: Volcano plots of VO_2peak_-associated plasma metabolite patterns, File S6: Calculation, File S7: Results, File S8: Output, File S9: Supplement, Table S1: Dataset including metabolite data and metadata, Table S2: Results of bi- and multivariate association analyses, Table S3: Results of multiple linear regression analyses.

## Abbreviations

AA	amino acid
AEE	activity energy expenditure
BP	blood pressure
BMC	bone mineral content
BMI	body mass index
CIs	confidence intervals
CRF	cardiorespiratory fitness
FEV1	forced expiratory pressure in one second
FM	fat mass
GC	gas chromatography
GC × GC	two-dimensional gas chromatography
Hb	hemoglobin
HDL	high-density lipoprotein
HEI-NVS	Healthy Eating Index (modified version)
HR_rest_	resting heart rate
KarMeN	Karlsruhe Metabolomics and Nutrition
LBM	lean body mass
LC	liquid chromatography
LCFA	long-chain fatty acid
LDL	low-density lipoprotein
LysoPC	lyso-phosphatidylcholine
MET	metabolic equivalent of task
MS	mass spectrometry
NMR	nuclear magnetic resonance
PA	physical activity
PC	phosphatidylcholine
PC aa	diacyl-phosphatidylcholine
PC ae	acyl-alkyl-phosphatidylcholine
PE	physical exercise
PF	physical fitness
PLS	partial least squares
PWV	pulse wave velocity
q	quarter
QC	quality control
r	Pearson correlation coefficient
R^2^	coefficient of determination
R^2^ (adjusted)	adjusted coefficient of determination
RMSE	root mean square error
SM	sphingomyelin
TCA	tricarboxylic acid
TGs	triglycerides
U	unknown
VATM	visceral adipose tissue mass
VC_max_	maximal vital capacity
VdW	Van der Waerden
VO_2max_	maximal oxygen uptake
VO_2peak_	peak oxygen uptake
