# Supplementary material for: Sex-Specific Relationship between the Cardiorespiratory Fitness and Plasma Metabolite Patterns in Healthy Humans—Results of the KarMeN Study
Source: Metabolites. 2021 Jul 17;11(7):463. doi: 10.3390/metabo11070463 (PMC8303204; doi:10.3390/metabo11070463)
Supplement: Supplementary file 1 [file metabolites-11-00463-s001.zip › File S1_Basic characteristics of the KarMeN participants according to VO2peak quarters.pdf]

File S1: Basic characteristics of the KarMeN participants according to VO_{2peak} quarters

Table 1. Characteristics of the female KarMeN participants (n=102) according to VO_{2peak} quarters.

Characteristics	VO_{2peak} Quarters				p*
	1st q (n=25)	2nd q (n=26)	3rd q (n=26)	4th q (n=25)	
**VO_{2peak} (mL kg^{-1} min^{-1})**	21.61 ± 2.14	27.50 ± 1.66	32.89 ± 1.48	40.91 ± 2.94	**<.0001**
**Age (years)**	62.26 ± 9.64	57.10 ± 11.05	50.28 ± 10.92	33.71 ± 9.58	**<.0001**
**Pre-/Post-Menopausal State [Δ]**	2/23	7/19	15/11	22/3	**<.0001**
**Weight (kg)**	68.88 ± 9.11	66.19 ± 7.23	60.97 ± 6.18	60.96 ± 7.00	**0.0008**
**Height (cm)**	164.16 ± 5.92	166.73 ± 6.63	166.79 ± 5.24	169.60 ± 7.21	**0.0484**
**BMI (kg/m²)**	25.60 ± 3.43	23.79 ± 1.92	21.92 ± 2.00	21.16 ± 1.79	**<.0001**
**LBM (kg)**	39.36 ± 4.30	40.60 ± 3.52	40.20 ± 3.06	41.10 ± 4.51	0.5504
**FM (%)**	39.24 ± 5.15	35.12 ± 4.84	30.33 ± 4.92	28.68 ± 4.33	**<.0001**
**VATM (kg)**	0.81 ± 0.48	0.42 ± 0.25	0.25 ± 0.22	0.12 ± 0.10	**<.0001**
**BMC (kg)**	2.24 ± 0.24	2.31 ± 0.38	2.34 ± 0.25	2.46 ± 0.30	0.0567
**Hb (g dL^{-1})**	13.72 ± 0.80	13.53 ± 0.68	13.46 ± 0.59	13.33 ± 0.87	0.408
**Glucose (mg dL^{-1})**	89.64 ± 8.14	85.27 ± 7.59	82.42 ± 6.47	82.04 ± 7.28	**0.0034**
**Insulin (µlU mL^{-1})**	10.32 ± 4.49	9.03 ± 2.96	8.53 ± 3.42	10.52 ± 4.47	0.2199
**HbA1c (%)**	5.47 ± 0.30	5.61 ± 0.37	5.42 ± 0.29	5.19 ± 0.44	**0.0065**
**TGs (mg dL^{-1})**	102.24 ± 35.38	85.04 ± 32.93	64.96 ± 18.12	62.72 ± 14.95	**<.0001**
**HDL cholesterol (mg dL^{-1})**	69.68 ± 12.98	79.15 ± 17.16	82.19 ± 18.84	79.36 ± 13.78	**0.0198**
**LDL cholesterol (mg dL^{-1})**	145.00 ± 30.37	137.35 ± 32.86	123.08 ± 32.04	104.24 ± 27.54	**<.0001**
**HR_{rest} (1 min^{-1})**	67.56 ± 7.83	66.23 ± 7.56	64.19 ± 10.14	64.84 ± 8.58	0.5298
**BP systolic (mmHg)**	128.44 ± 16.57	124.85 ± 16.93	113.50 ± 12.90	106.72 ± 7.17	**<.0001**
**BP diastolic (mmHg)**	88.68 ± 9.22	86.04 ± 8.53	78.65 ± 10.51	74.52 ± 8.21	**<.0001**
**PWV (m s^{-1}) °**	9.73 ± 1.76	9.28 ± 2.20	7.69 ± 1.16	6.64 ± 0.81	**<.0001**
**VC_{max} (L)**	3.25 ± 0.46	3.61 ± 0.54	3.66 ± 0.42	4.04 ± 0.68	**<.0001**
**FEV1 (L)**	2.47 ± 0.40	2.75 ± 0.40	2.79 ± 0.42	3.36 ± 0.63	**<.0001**
**AEE (kcal d^{-1})**	639.36 ± 199.65	782.77 ± 296.70	754.42 ± 185.38	777.48 ± 257.39	0.0814
**Total MET (MET-min week^{-1})**	6469.8 ± 4065.7	7032.8 ± 4450.5	6518.1 ± 3337.5	3920.3 ± 2099.1	**0.0008**
**HEI-NVS**	73.86 ± 9.62	69.82 ± 9.99	76.15 ± 10.38	73.24 ± 8.38	0.1805

Based on the VO_{2peak} quartiles in the female subgroup, the VO_{2peak} data were divided into four quarters (q) and basic characteristics of the subgroups of the corresponding females of the quarters are presented. Data are given in mean ± SD. [Δ]: number of female participants in the pre-/post-menopausal state; ° n=23 (1st q), n=25 (2nd q). *: significant differences between quarters according to Welch ANOVA (Chi² test) for numeric (categorical) variables. AEE: activity energy expenditure; BMC: bone mineral content; BMI: body mass index; BP: blood pressure; FEV1: forced expiratory pressure in one second; FM: fat mass; Hb: hemoglobin; HDL: high-density lipoprotein; HEI-NVS: Healthy Eating Index (modified version); HR_{rest}: resting heart rate; LBM: lean body mass; LDL: low-density lipoprotein; MET: metabolic equivalent of task; PWV: pulse wave velocity; TGs: triglycerides; VATM: visceral adipose tissue mass; VC_{max}: maximal vital capacity; VO_{2peak}: peak oxygen uptake.

Table 2. Characteristics of the male KarMeN participants (n=150) according to VO_{2peak} quarters.

Characteristics	VO_{2peak} Quarters				p*
	1st q (n=37)	2nd q (n=38)	3rd q (n=38)	4th q (n=37)	
**VO_{2peak} (mL kg^{-1} min^{-1})**	30.77 ± 4.04	40.32 ± 2.15	48.53 ± 2.26	58.45 ± 4.71	**<.0001**
**Age (years)**	59.02 ± 14.37	45.12 ± 17.27	38.40 ± 13.69	27.62 ± 7.76	**<.0001**
**Weight (kg)**	83.17 ± 10.35	79.42 ± 10.50	76.61 ± 9.85	75.15 ± 7.64	**0.0025**
**Height (cm)**	177.39 ± 7.24	180.74 ± 6.18	181.51 ± 8.99	180.89 ± 6.32	**0.0809**
**BMI (kg/m²)**	26.40 ± 2.59	24.27 ± 2.63	23.20 ± 2.10	22.98 ± 2.16	**<.0001**
**LBM (kg)**	56.14 ± 5.95	57.69 ± 6.60	57.74 ± 6.85	60.45 ± 6.76	**0.0449**
**FM (%)**	28.80 ± 3.92	23.69 ± 5.17	20.77 ± 4.72	16.07 ± 4.19	**<.0001**
**VATM (kg)**	1.70 ± 0.74	0.77 ± 0.55	0.50 ± 0.44	0.26 ± 0.15	**<.0001**
**BMC (kg)**	3.19 ± 0.41	3.11 ± 0.42	3.27 ± 0.45	3.27 ± 0.44	0.3633
**Hb (g dL^{-1})**	15.11 ± 1.15	14.95 ± 0.80	15.01 ± 0.85	15.07 ± 0.75	0.8748
**Glucose (mg dL^{-1})**	90.57 ± 9.73	86.63 ± 7.95	84.50 ± 6.55	83.35 ± 7.52	**0.004**
**Insulin (µlU mL^{-1})**	10.86 ± 4.36	10.99 ± 6.52	9.06 ± 3.10	9.49 ± 2.86	0.1293
**HbA1c (%)**	5.54 ± 0.34	5.37 ± 0.33	5.26 ± 0.28	5.25 ± 0.32	**0.0012**
**TGs (mg dL^{-1})**	119.92 ± 45.84	100.95 ± 60.22	82.11 ± 37.49	81.05 ± 29.25	**0.0002**
**HDL cholesterol (mg dL^{-1})**	58.54 ± 13.51	61.32 ± 13.70	68.61 ± 16.00	62.68 ± 11.38	**0.0376**
**LDL cholesterol (mg dL^{-1})**	150.68 ± 43.95	117.74 ± 41.03	114.18 ± 33.45	97.65 ± 28.05	**<.0001**
**HR_{rest} (1 min^{-1})**	64.14 ± 10.35	63.26 ± 7.80	57.16 ± 7.58	55.95 ± 8.21	**<.0001**
**BP systolic (mmHg)**	133.41 ± 14.79	126.32 ± 14.42	126.08 ± 11.16	121.95 ± 10.96	**0.0044**
**BP diastolic (mmHg)**	90.97 ± 9.59	85.21 ± 9.21	82.53 ± 7.17	76.30 ± 8.62	**<.0001**
**PWV (m s^{-1}) [v]**	8.39 ± 1.49	7.23 ± 1.30	6.74 ± 0.85	6.17 ± 0.64	**<.0001**
**VC_{max} (L)**	4.89 ± 0.90	5.35 ± 0.87	5.61 ± 0.85	5.80 ± 0.72	**<.0001**
**FEV1 (L)**	3.63 ± 0.75	4.20 ± 0.80	4.39 ± 0.76	4.68 ± 0.66	**<.0001**
**AEE (kcal d^{-1}) [vv]**	798.69 ± 499.28	1017.03 ± 562.34	1193.26 ± 483.33	1546.89 ± 625.66	**<.0001**
**Total MET (MET-min week^{-1})**	6322.9 ± 5689.9	5985.5 ± 5072.6	4769.2 ± 3598.5	5921.7 ± 3937.6	0.3929
**HEI-NVS**	70.14 ± 10.97	72.15 ± 9.84	69.13 ± 8.78	72.39 ± 9.96	0.3828

Based on the VO_{2peak} quartiles in the male subgroup, the VO_{2peak} data were divided into four quarters (q) and basic characteristics of the subgroups of the corresponding males of the quarters are presented. Data are given in mean ± SD. [v] n=36 (1st q); [vv] n=35 (1st q), n=37 (2nd q). *: significant differences between quarters according to Welch ANOVA. AEE: activity energy expenditure; BMC: bone mineral content; BMI: body mass index; BP: blood pressure; FEV1: forced expiratory pressure in one second; FM: fat mass; Hb: hemoglobin; HDL: high-density lipoprotein; HEI-NVS: Healthy Eating Index (modified version); HR_{rest}: resting heart rate; LBM: lean body mass; LDL: low-density lipoprotein; MET: metabolic equivalent of task; PWV: pulse wave velocity; TGs: triglycerides; VATM: visceral adipose tissue mass; VC_{max}: maximal vital capacity; VO_{2peak}: peak oxygen uptake.
