# Supplementary material for: Sex-Specific Relationship between the Cardiorespiratory Fitness and Plasma Metabolite Patterns in Healthy Humans—Results of the KarMeN Study
Source: Metabolites. 2021 Jul 17;11(7):463. doi: 10.3390/metabo11070463 (PMC8303204; doi:10.3390/metabo11070463)
Supplement: Supplementary file 1 [file metabolites-11-00463-s001.zip › File S2_Graphical overview of associations between the VO2peak and plasma metabolites.pdf]

File S2: Graphical overview of associations between the VO2peak and plasma metabolites

Partial correlations: Direction and strength of bivariate associations between the VO2peak and each metabolites, expressed as partial Pearson correlation coefficients with 95% confidence intervals of Van der Waerden-transformed variables.
Contribution to PLS-model: Importance of contribution of each metabolite to the multivariate association in the obtained PLS models with the VO2peak, expressed as the negative logarithm of relative frequencies of permutation-obtained rank products below measured rank products.

F*	Females adjusted for age and menopausal status
F**	Females adjusted for age, menopausal status and the 21 phenotypical and clinical parameters
M*	Males adjusted for age
M**	Males adjusted for age and the 21 phenotypical and clinical parameters

Major Metabolic Pathways:

Lipid metabolism	Amino acid metabolism	Xenobiotics and related metabolism
Energy metabolism	Carbohydrate metabolism	Mammalian-microbial cometabolism
Nucleotide metabolism	Cofactors and vitamins metabolism	Unknown

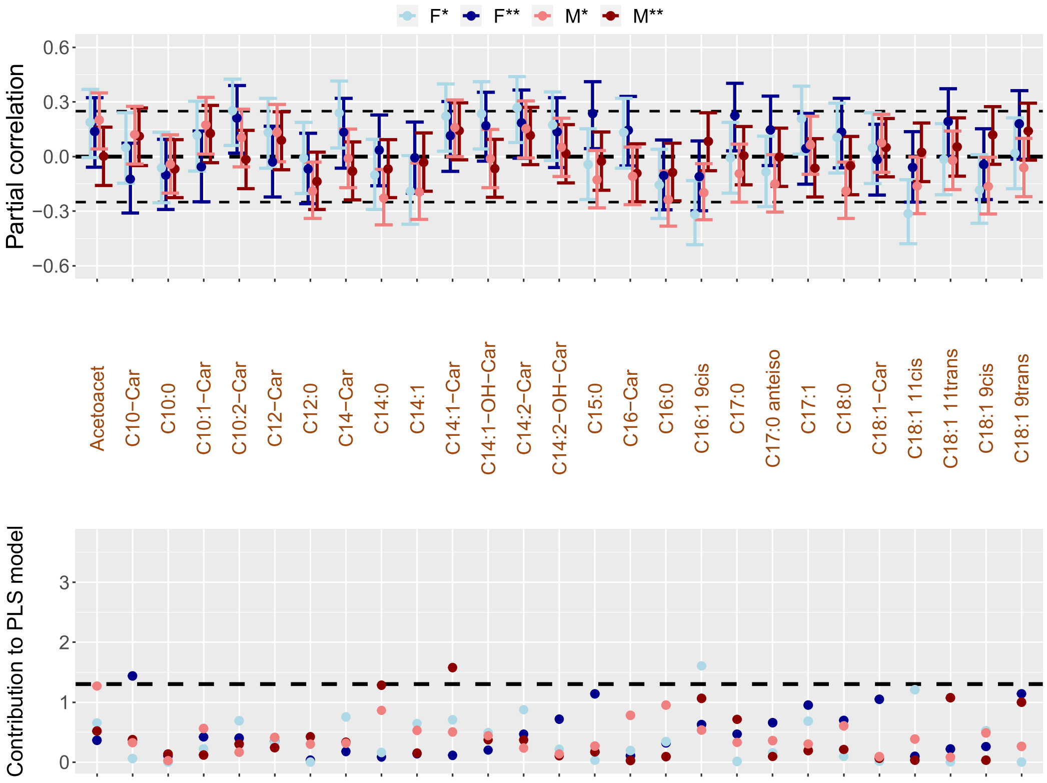

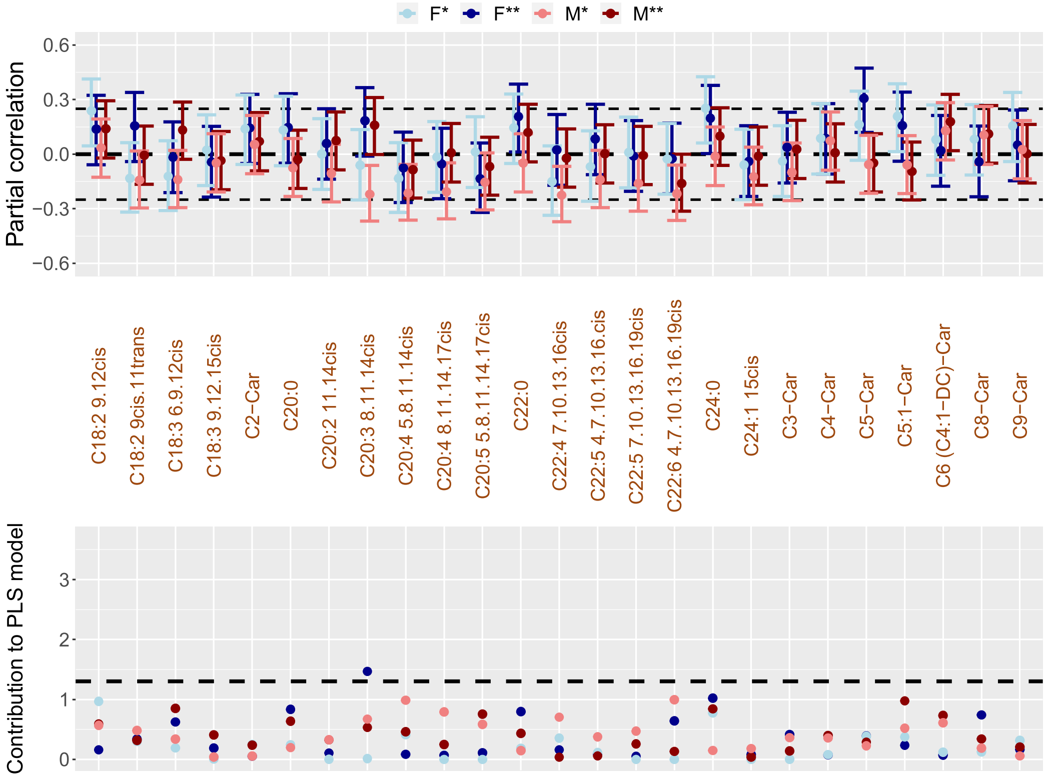

F*
F**
M*
M**
Partial correlation
0.6
0.3
0.0
−0.3
−0.6
C18:2 9.12cis
C18:2 9cis.11trans
C18:3 6.9.12cis
C18:3 9.12.15cis
C2−Car
C20:0
C20:2 11.14cis
C20:3 8.11.14cis
C20:4 5.8.11.14cis
C20:4 8.11.14.17cis
C20:5 5.8.11.14.17cis
C22:0
C22:4 7.10.13.16cis
C22:5 4.7.10.13.16.cis
C22:5 7.10.13.16.19cis
C22:6 4.7.10.13.16.19cis
C24:0
C24:1 15cis
C3−Car
C4−Car
C5−Car
C5:1−Car
C6 (C4:1−DC)−Car
C8−Car
C9−Car
Contribution to PLS model
3
2
1
0

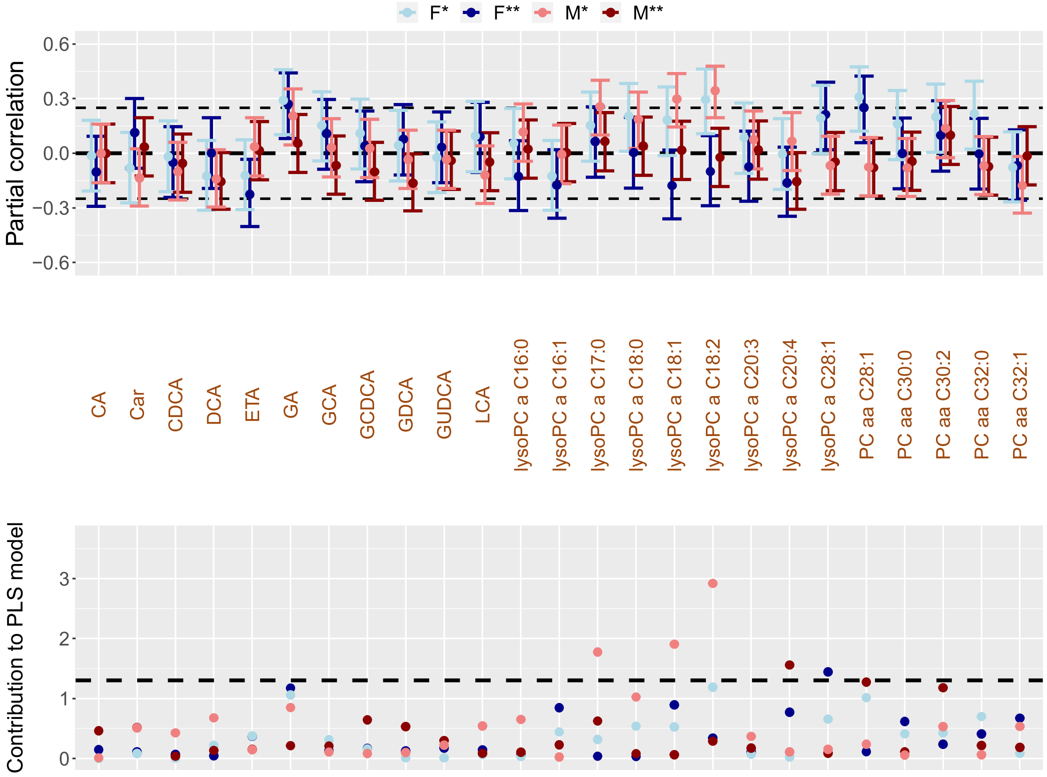

F*
F**
M*
M**
Partial correlation
0.6
0.3
0.0
−0.3
−0.6
CA
Car
CDCA
DCA
ETA
GA
GCA
GCDCA
GDCA
GUDCA
LCA
lysoPC a C16:0
lysoPC a C16:1
lysoPC a C17:0
lysoPC a C18:0
lysoPC a C18:1
lysoPC a C18:2
lysoPC a C20:3
lysoPC a C20:4
lysoPC a C28:1
PC aa C28:1
PC aa C30:0
PC aa C30:2
PC aa C32:0
PC aa C32:1
Contribution to PLS model
3
2
1
0

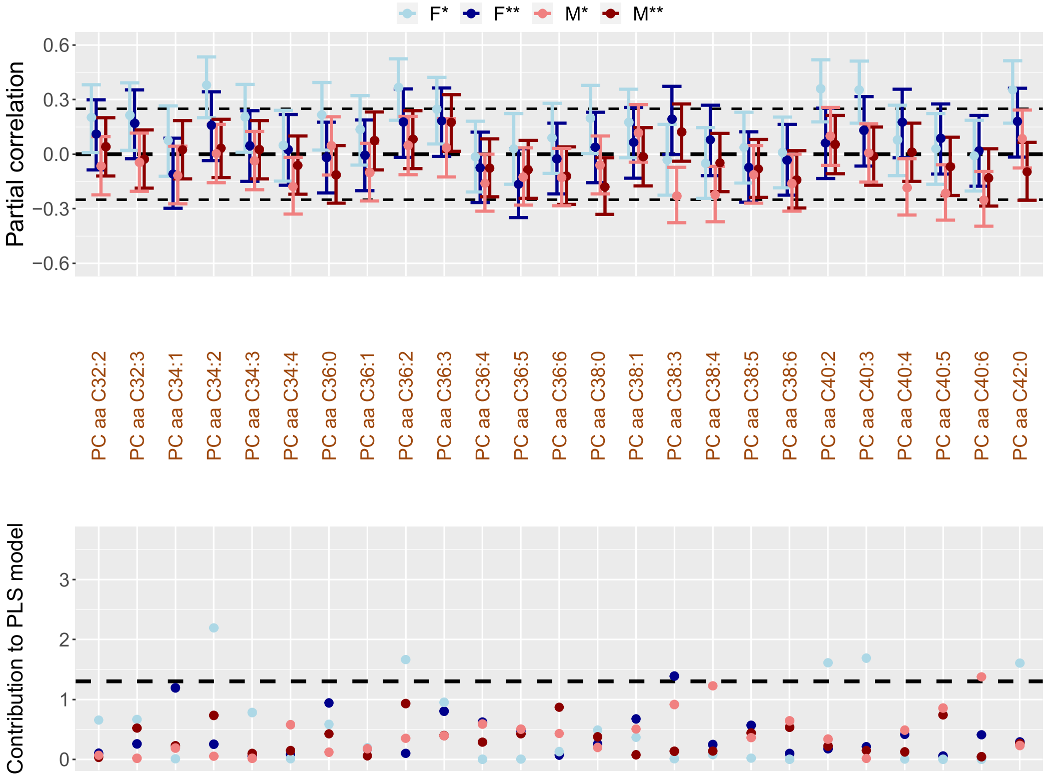
F* F** M* M**
Partial correlation
0.6
0.3
0.0
−0.3
−0.6
PC aa C32:2
PC aa C32:3
PC aa C34:1
PC aa C34:2
PC aa C34:3
PC aa C34:4
PC aa C36:0
PC aa C36:1
PC aa C36:2
PC aa C36:3
PC aa C36:4
PC aa C36:5
PC aa C36:6
PC aa C38:0
PC aa C38:1
PC aa C38:3
PC aa C38:4
PC aa C38:5
PC aa C38:6
PC aa C40:2
PC aa C40:3
PC aa C40:4
PC aa C40:5
PC aa C40:6
PC aa C42:0
Contribution to PLS model
3
2
1
0

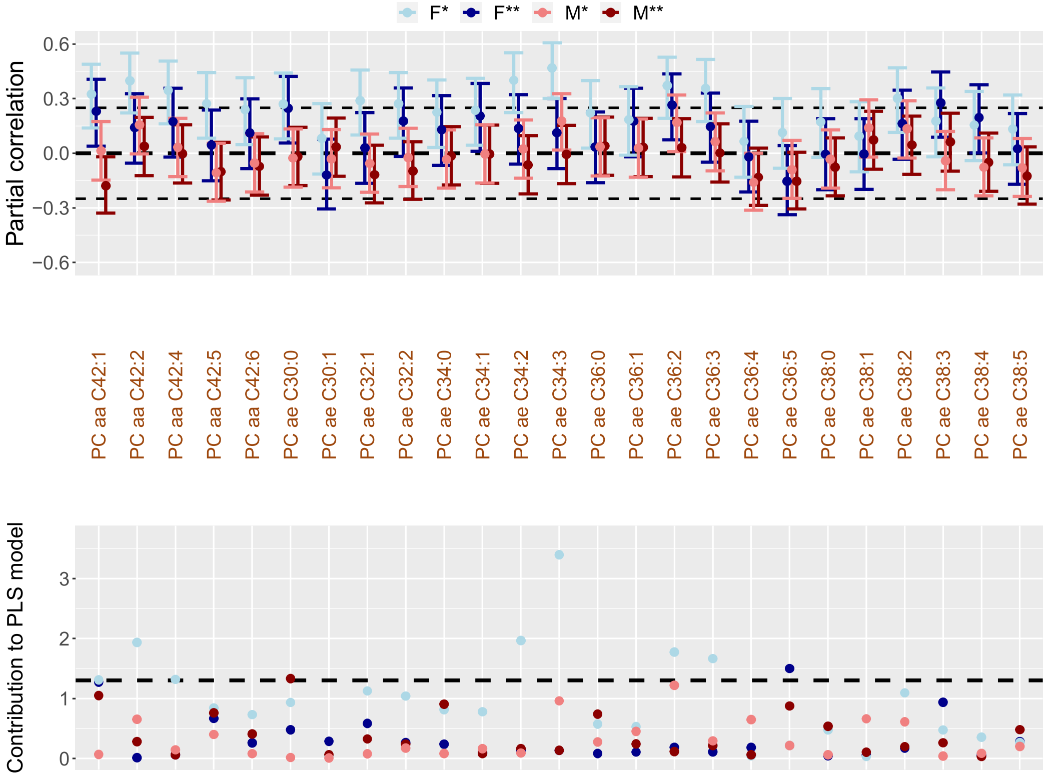
F* F** M* M**
Partial correlation
0.6
0.3
0.0
−0.3
−0.6
PC aa C42:1
PC aa C42:2
PC aa C42:4
PC aa C42:5
PC aa C42:6
PC ae C30:0
PC ae C30:1
PC ae C32:1
PC ae C32:2
PC ae C34:0
PC ae C34:1
PC ae C34:2
PC ae C34:3
PC ae C36:0
PC ae C36:1
PC ae C36:2
PC ae C36:3
PC ae C36:4
PC ae C36:5
PC ae C38:0
PC ae C38:1
PC ae C38:2
PC ae C38:3
PC ae C38:4
PC ae C38:5
Contribution to PLS model
3
2
1
0

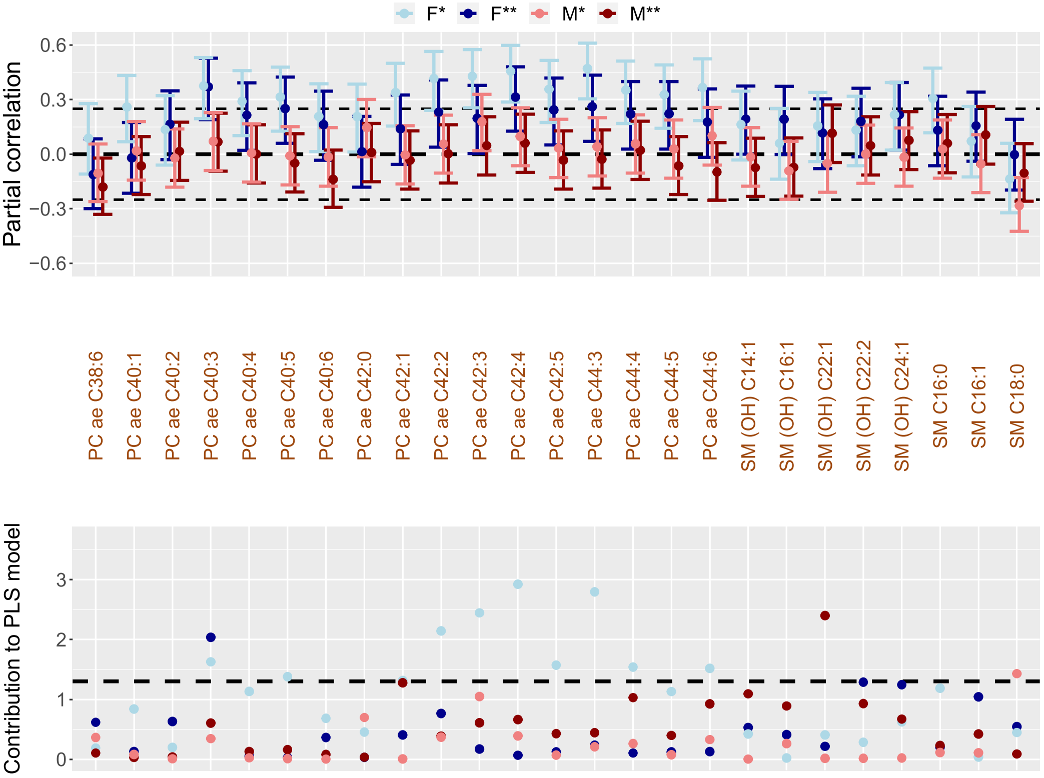
F*
F**
M*
M**
Partial correlation
0.6
0.3
0.0
−0.3
−0.6
PC ae C38:6
PC ae C40:1
PC ae C40:2
PC ae C40:3
PC ae C40:4
PC ae C40:5
PC ae C40:6
PC ae C42:0
PC ae C42:1
PC ae C42:2
PC ae C42:3
PC ae C42:4
PC ae C42:5
PC ae C44:3
PC ae C44:4
PC ae C44:5
PC ae C44:6
SM (OH) C14:1
SM (OH) C16:1
SM (OH) C22:1
SM (OH) C22:2
SM (OH) C24:1
SM C16:0
SM C16:1
SM C18:0
Contribution to PLS model
3
2
1
0

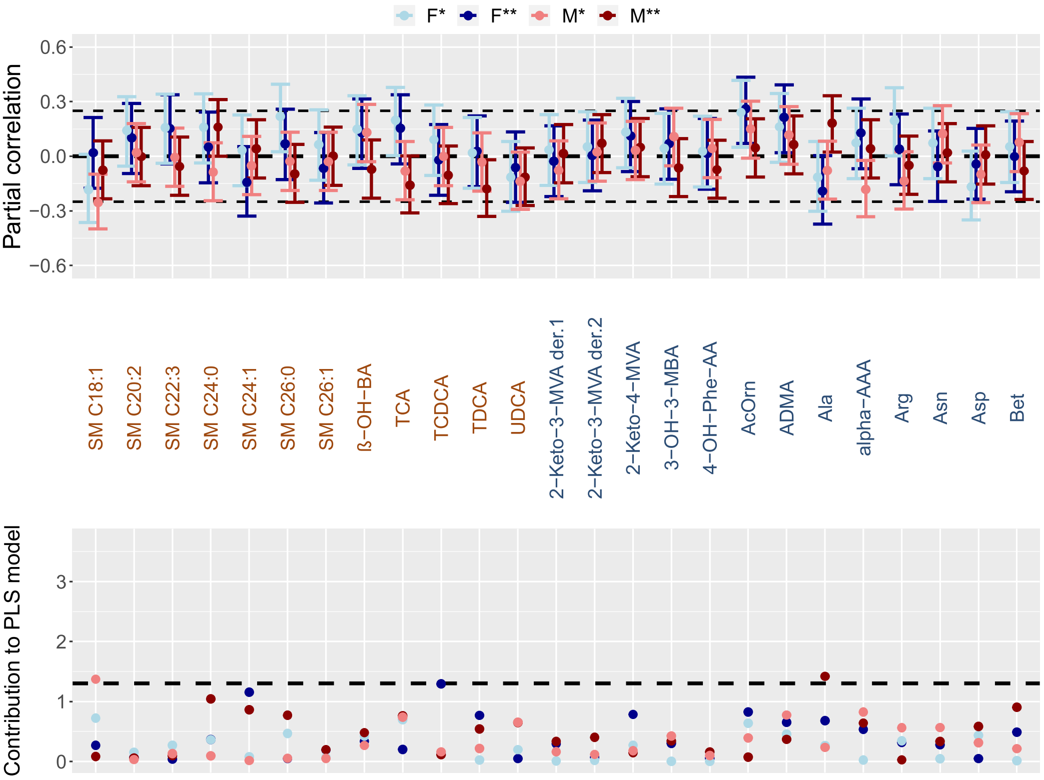
F*
F**
M*
M**
Partial correlation
0.6
0.3
0.0
−0.3
−0.6
SM C18:1
SM C20:2
SM C22:3
SM C24:0
SM C24:1
SM C26:0
SM C26:1
ß−OH−BA
TCA
TCDCA
TDCA
UDCA
2−Keto−3−MVA der.1
2−Keto−3−MVA der.2
2−Keto−4−MVA
3−OH−3−MBA
4−OH−Phe−AA
AcOrn
ADMA
Ala
alpha−AAA
Arg
Asn
Asp
Bet
Contribution to PLS model
3
2
1
0

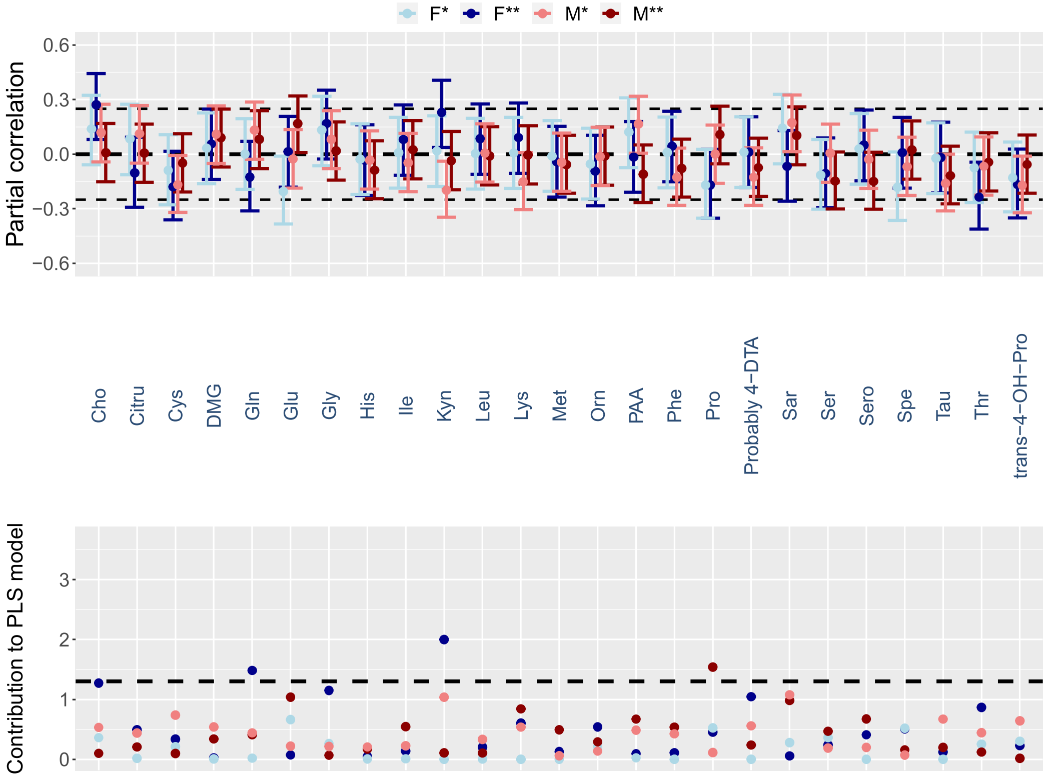
F*
F**
M*
M**
Partial correlation
0.6
0.3
0.0
−0.3
−0.6
Cho
Citru
Cys
DMG
Gln
Glu
Gly
His
Ile
Kyn
Leu
Lys
Met
Orn
PAA
Phe
Pro
Probably 4−DTA
Sar
Ser
Sero
Spe
Tau
Thr
trans−4−OH−Pro
Contribution to PLS model
3
2
1
0

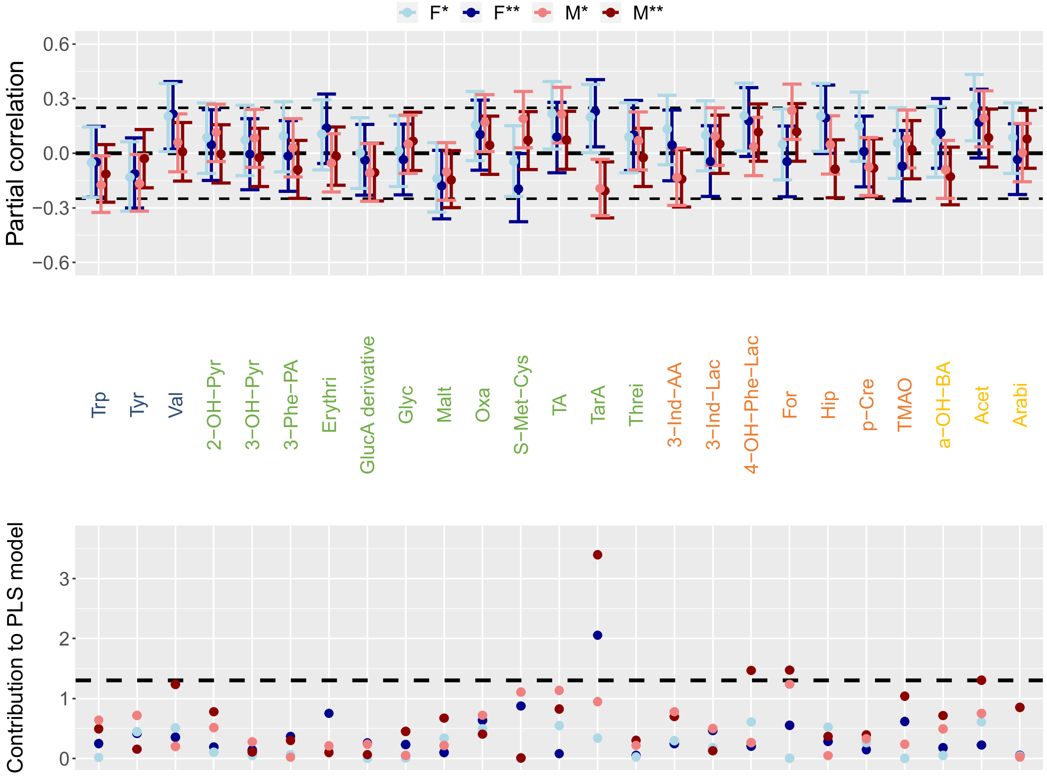
F*
F**
M*
M**
Partial correlation
0.6
0.3
0.0
−0.3
−0.6
Trp
Tyr
Val
2−OH−Pyr
3−OH−Pyr
3−Phe−PA
Erythri
GlucA derivative
Glyc
Malt
Oxa
S−Met−Cys
TA
TarA
Threi
3−Ind−AA
3−Ind−Lac
4−OH−Phe−Lac
For
Hip
p−Cre
TMAO
a−OH−BA
Acet
Arabi
Contribution to PLS model
3
2
1
0

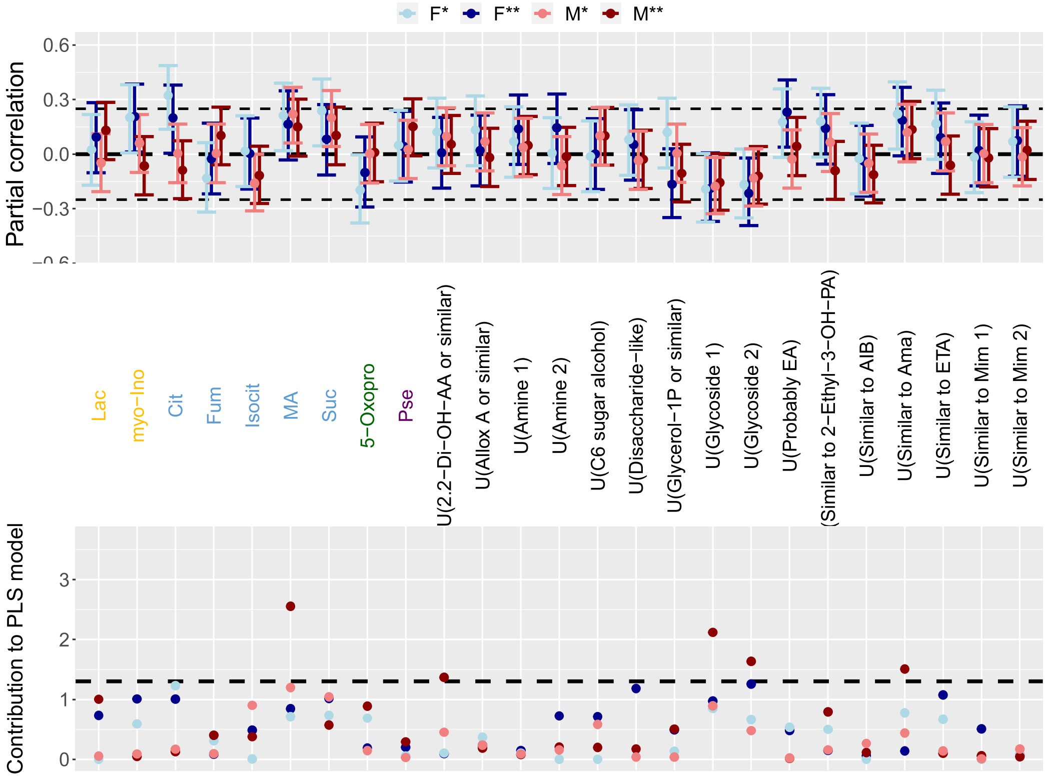
F*
F**
M*
M**
Partial correlation
Contribution to PLS model
Lac
myo-Ino
Cit
Fum
Isocit
MA
Suc
5-Oxopro
Pse
U(2.2-Di-OH-AA or similar)
U(Allox A or similar)
U(Amine 1)
U(Amine 2)
U(C6 sugar alcohol)
U(Disaccharide-like)
U(Glycerol-1P or similar)
U(Glycoside 1)
U(Glycoside 2)
U(Probably EA)
(Similar to 2-Ethyl-3-OH-PA)
U(Similar to AIB)
U(Similar to Ama)
U(Similar to ETA)
U(Similar to Mim 1)
U(Similar to Mim 2)

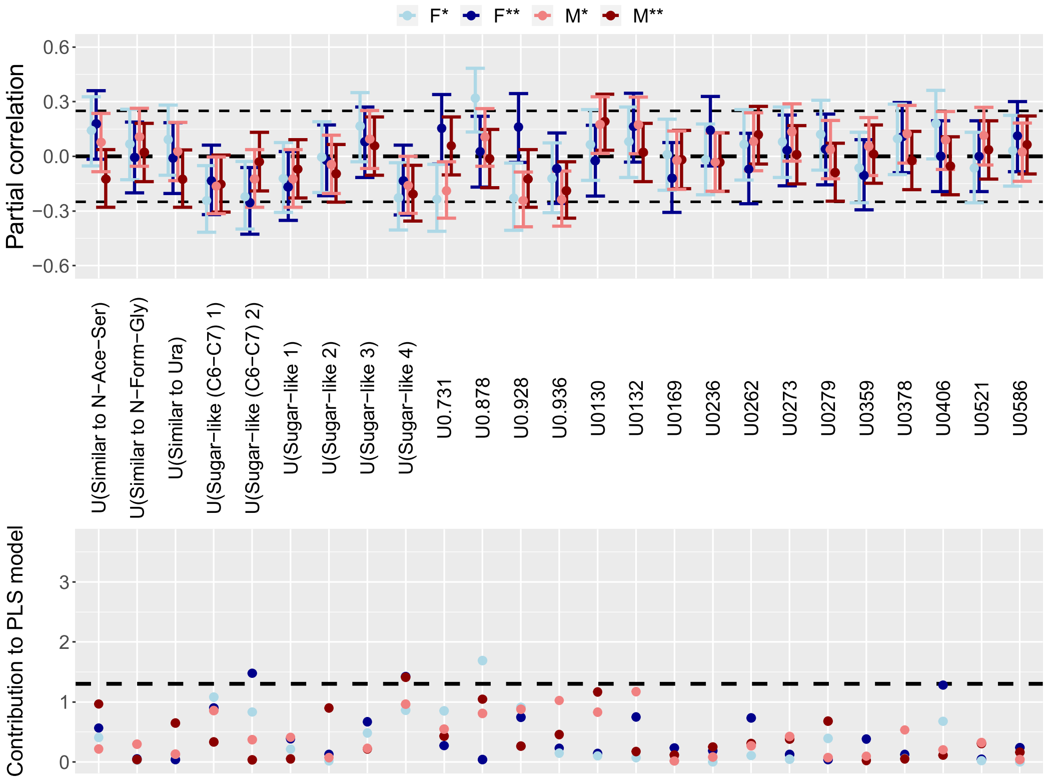
F*
F**
M*
M**
Partial correlation
Contribution to PLS model
U(Similar to N-Ace-Ser)
U(Similar to N-Form-Gly)
U(Similar to Ura)
U(Sugar-like (C6-C7) 1)
U(Sugar-like (C6-C7) 2)
U(Sugar-like 1)
U(Sugar-like 2)
U(Sugar-like 3)
U(Sugar-like 4)
U0.731
U0.878
U0.928
U0.936
U0130
U0132
U0169
U0236
U0262
U0273
U0279
U0359
U0378
U0406
U0521
U0586

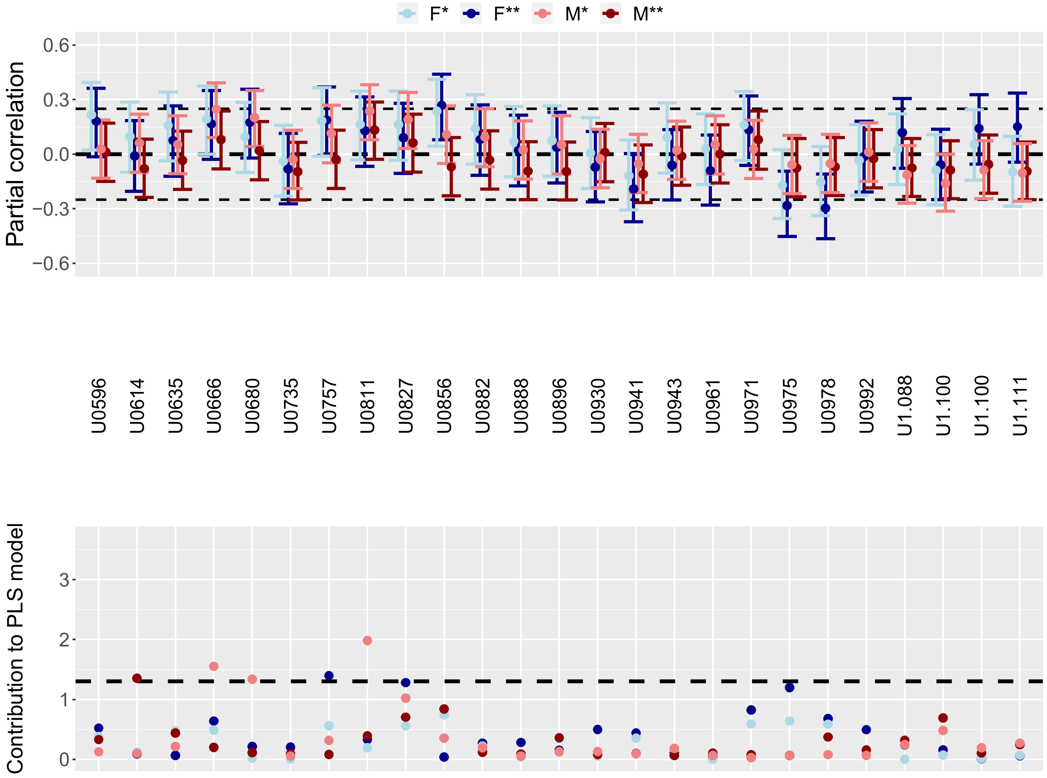
F*
F**
M*
M**
Partial correlation
0.6
0.3
0.0
−0.3
−0.6
U0596
U0614
U0635
U0666
U0680
U0735
U0757
U0811
U0827
U0856
U0882
U0888
U0896
U0930
U0941
U0943
U0961
U0971
U0975
U0978
U0992
U1.088
U1.100
U1.100
U1.111
Contribution to PLS model
3
2
1
0

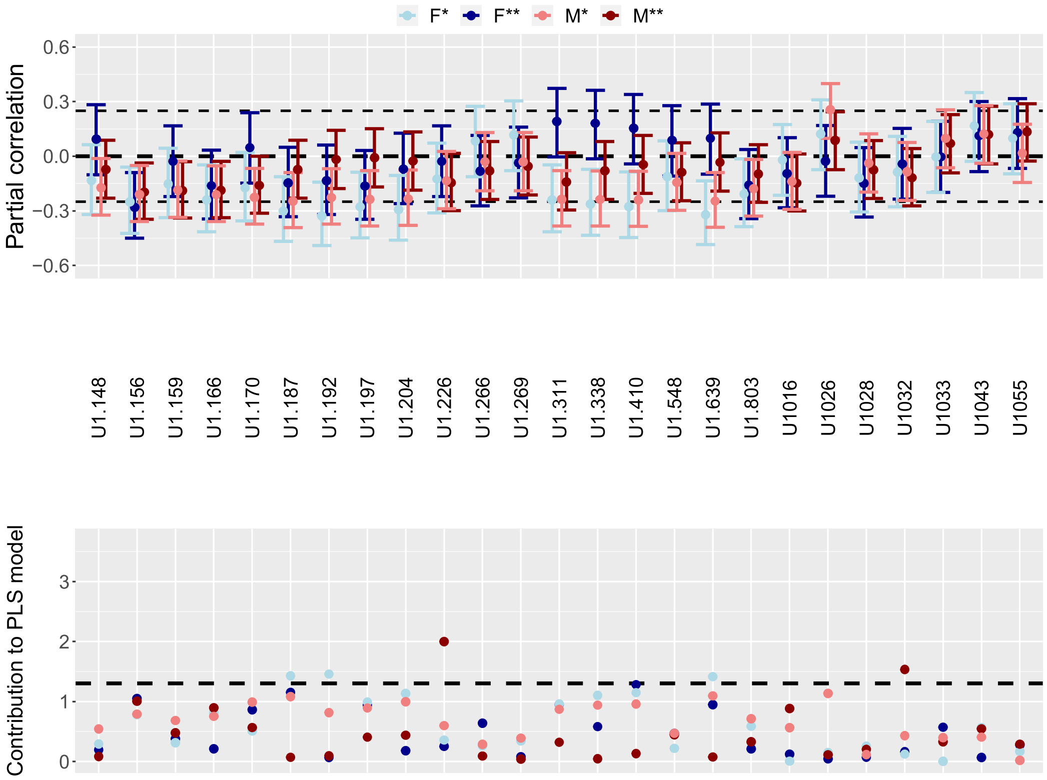
F*
F**
M*
M**
Partial correlation
0.6
0.3
0.0
−0.3
−0.6
U1.148
U1.156
U1.159
U1.166
U1.170
U1.187
U1.192
U1.197
U1.204
U1.226
U1.266
U1.269
U1.311
U1.338
U1.410
U1.548
U1.639
U1.803
U1016
U1026
U1028
U1032
U1033
U1043
U1055
Contribution to PLS model
3
2
1
0

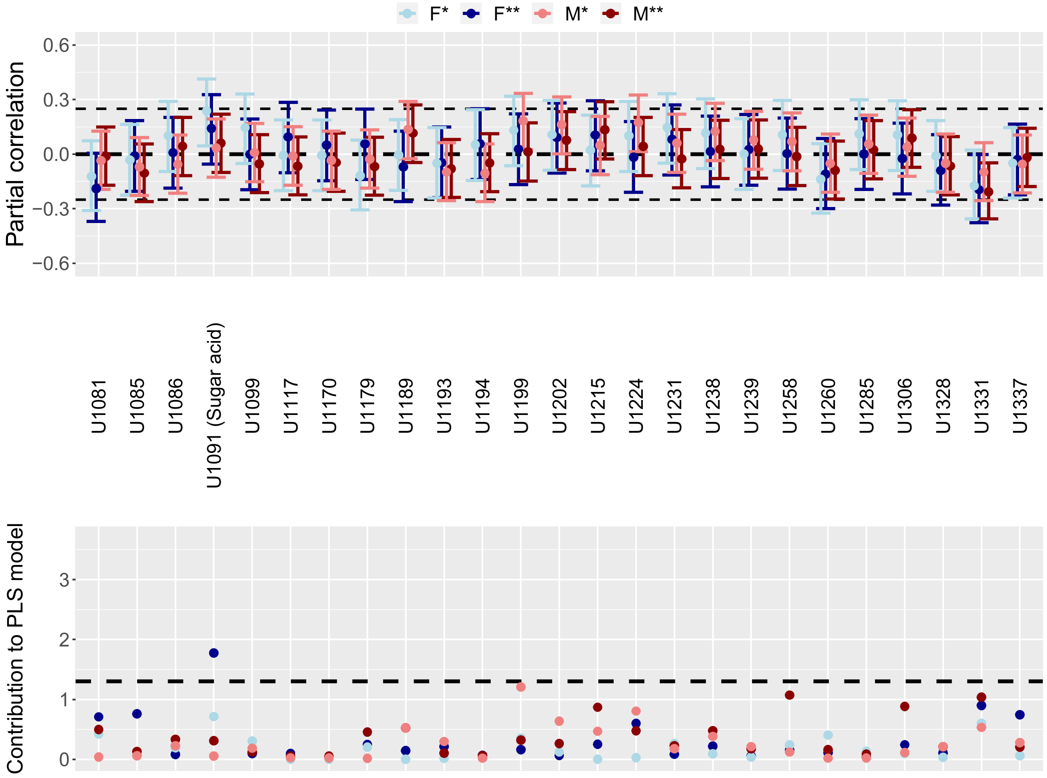

F*
F**
M*
M**
Partial correlation
0.6
0.3
0.0
−0.3
−0.6
U1081
U1085
U1086
U1091 (Sugar acid)
U1099
U1117
U1170
U1179
U1189
U1193
U1194
U1199
U1202
U1215
U1224
U1231
U1238
U1239
U1258
U1260
U1285
U1306
U1328
U1331
U1337
Contribution to PLS model
3
2
1
0

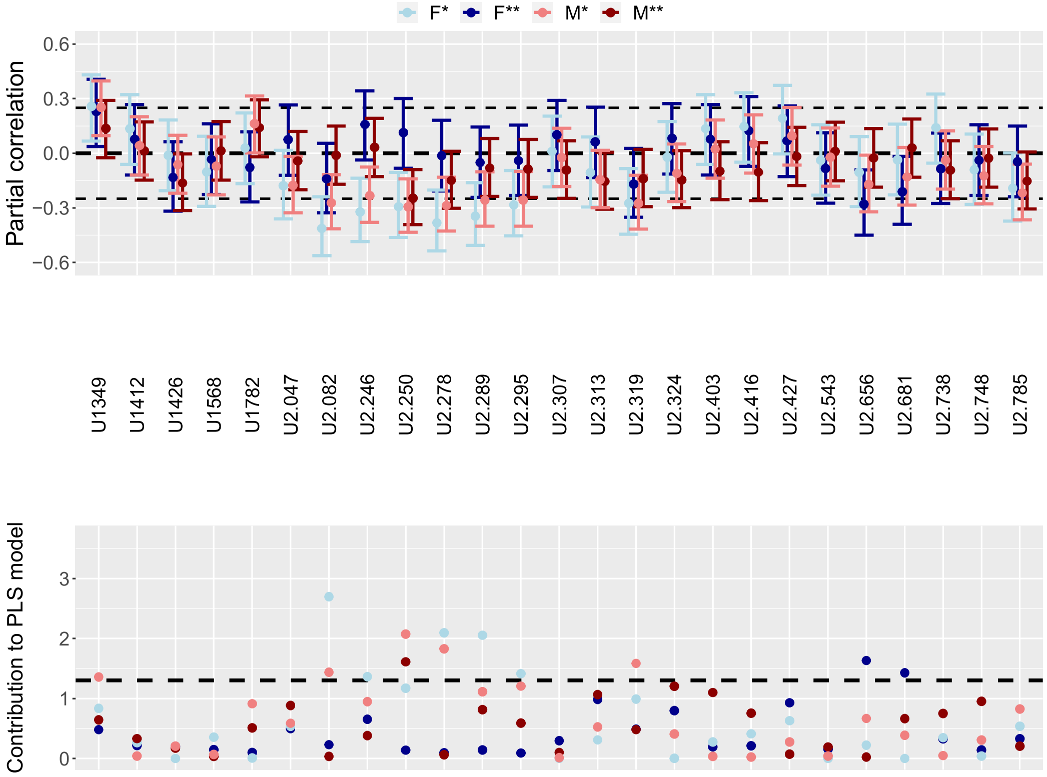

F*
F**
M*
M**
Partial correlation
0.6
0.3
0.0
−0.3
−0.6
U1349
U1412
U1426
U1568
U1782
U2.047
U2.082
U2.246
U2.250
U2.278
U2.289
U2.295
U2.307
U2.313
U2.319
U2.324
U2.403
U2.416
U2.427
U2.543
U2.656
U2.681
U2.738
U2.748
U2.785
Contribution to PLS model
3
2
1
0

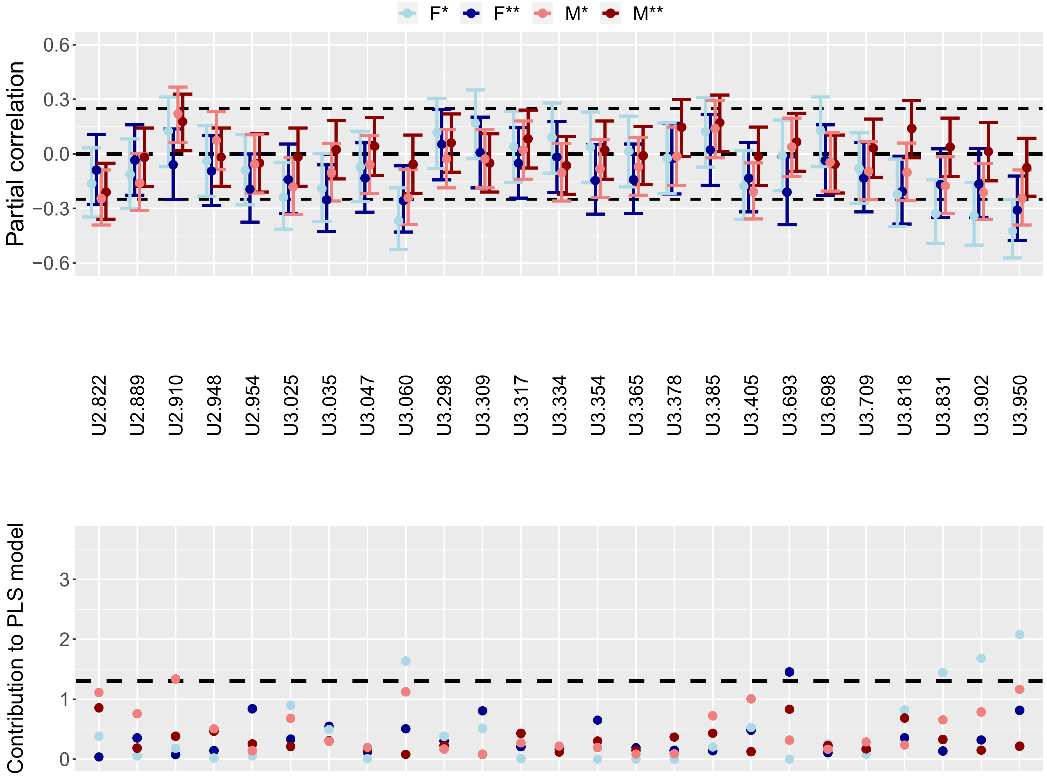
F* F** M* M**
Partial correlation
0.6
0.3
0.0
−0.3
−0.6
U2.822 U2.889 U2.910 U2.948 U2.954 U3.025 U3.035 U3.047 U3.060 U3.298 U3.309 U3.317 U3.334 U3.354 U3.365 U3.378 U3.385 U3.405 U3.693 U3.698 U3.709 U3.818 U3.831 U3.902 U3.950
Contribution to PLS model
3
2
1
0

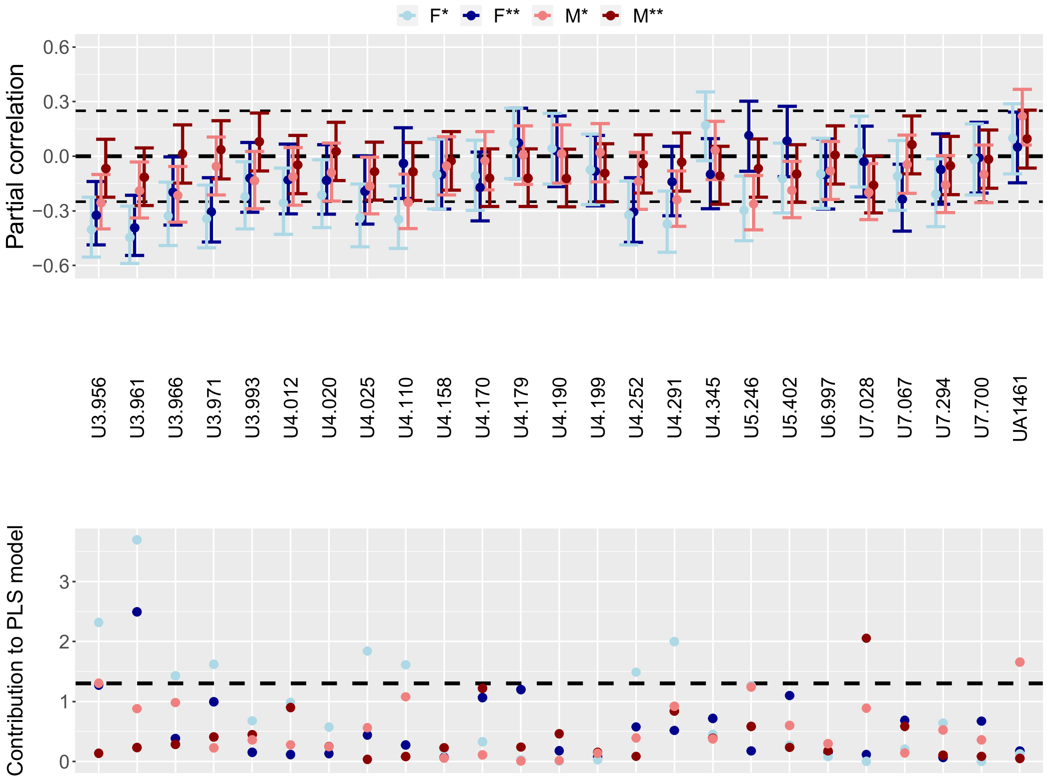
F* F** M* M**
Partial correlation
0.6
0.3
0.0
−0.3
−0.6
U3.956 U3.961 U3.966 U3.971 U3.993 U4.012 U4.020 U4.025 U4.110 U4.158 U4.170 U4.179 U4.190 U4.199 U4.252 U4.291 U4.345 U5.246 U5.402 U6.997 U7.028 U7.067 U7.294 U7.700 UA1461
Contribution to PLS model
3
2
1
0
