# Supplementary material for: Sex-Specific Relationship between the Cardiorespiratory Fitness and Plasma Metabolite Patterns in Healthy Humans—Results of the KarMeN Study
Source: Metabolites. 2021 Jul 17;11(7):463. doi: 10.3390/metabo11070463 (PMC8303204; doi:10.3390/metabo11070463)
Supplement: Supplementary file 1 [file metabolites-11-00463-s001.zip › File S4_Evaluation of PLS approaches.pdf]

File S4: Evaluation of PLS approaches

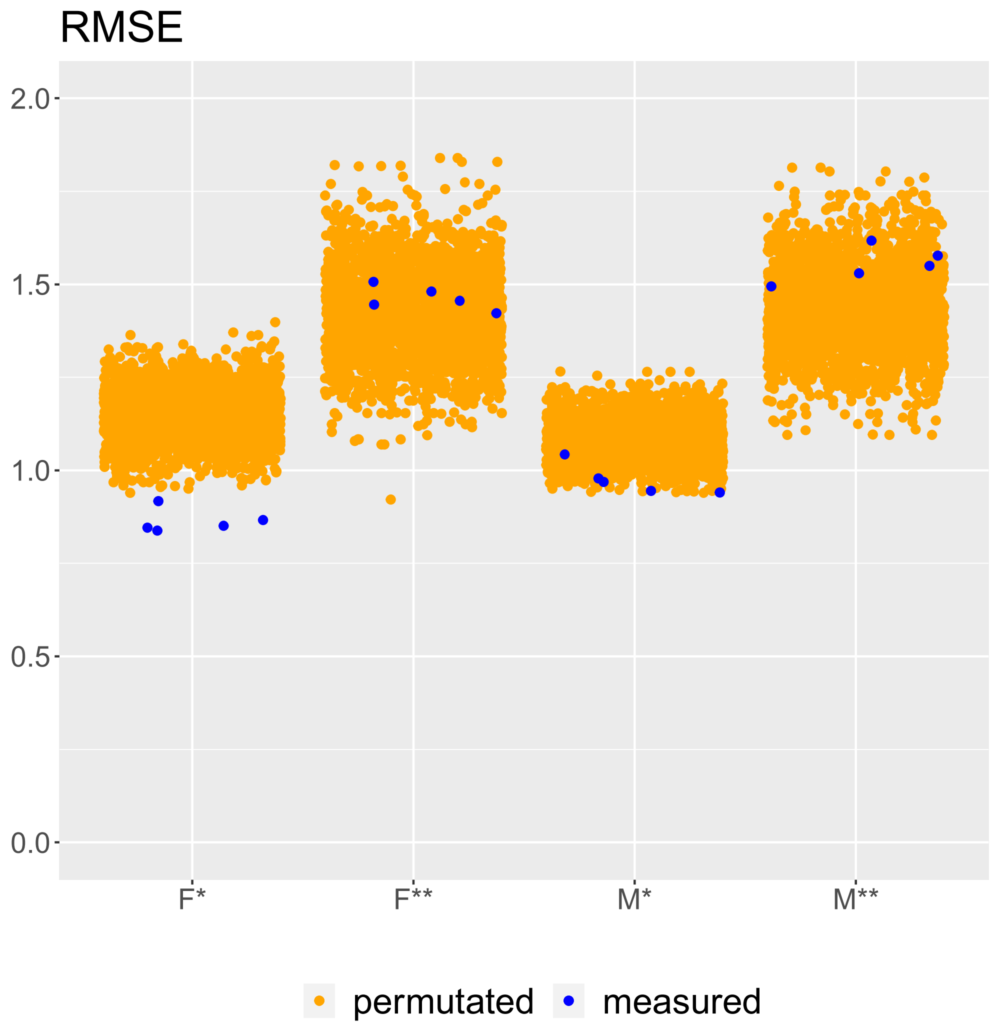

Mean of the root mean squares errors (RMSE) on the test samples across the 20 repetitions of the pls approaches. Blue: 5 repetitions for each subgroup using the original data. Orange: 2500 permutation for each subgroup.

F*	Females adjusted for age and menopausal status
F**	Females adjusted for age, menopausal status and the 21 phenotypical and clinical parameters
M*	Males adjusted for age
M**	Males adjusted for age and the 21 phenotypical and clinical parameters
