# Supplementary material for: Sex-Specific Relationship between the Cardiorespiratory Fitness and Plasma Metabolite Patterns in Healthy Humans—Results of the KarMeN Study
Source: Metabolites. 2021 Jul 17;11(7):463. doi: 10.3390/metabo11070463 (PMC8303204; doi:10.3390/metabo11070463)
Supplement: Supplementary file 1 [file metabolites-11-00463-s001.zip › File S5_Volcano plots of VO2peak-associated plasma metabolite patterns.pdf]

File S5 Volcano plots of VO2peak-associated plasma metabolite patterns

F*	Females adjusted for age and menopausal status
F**	Females adjusted for age, menopausal status and the 21 phenotypical and clinical parameters
M*	Males adjusted for age
M**	Males adjusted for age and the 21 phenotypical and clinical parameters

Major Metabolic Pathways:

Lipid metabolism	Amino acid metabolism	Xenobiotics and related metabolism
Energy metabolism	Carbohydrate metabolism	Mammalian-microbial cometabolism
Nucleotide metabolism	Cofactors and vitamins metabolism	Unknown

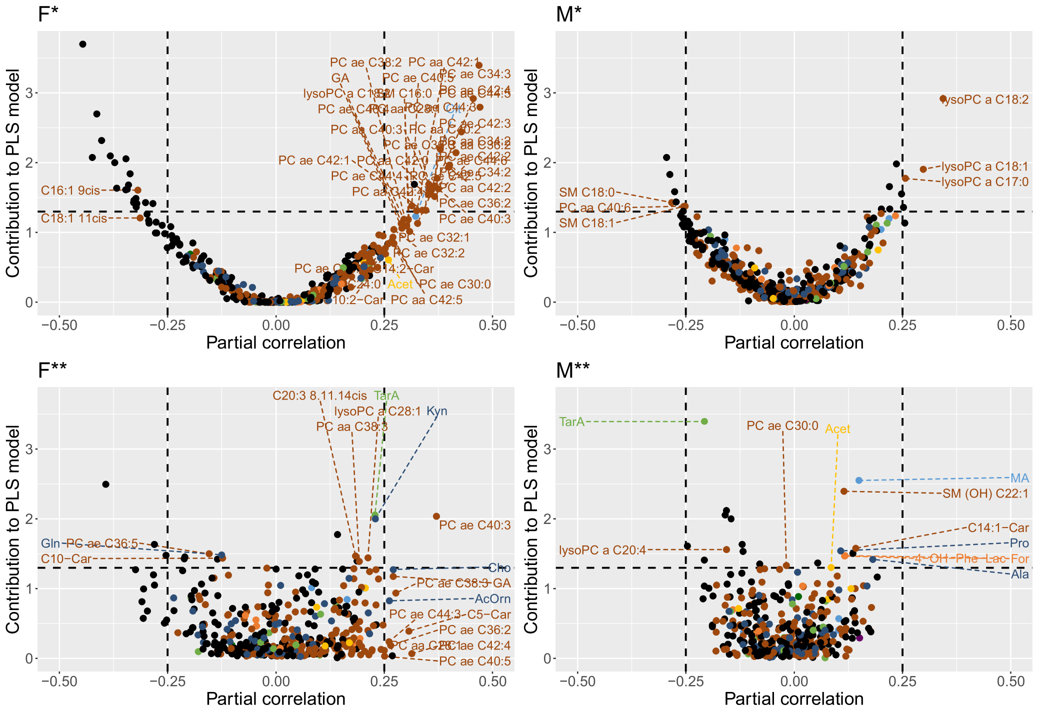

Volcano plots illustrating sex-specific plasma metabolite patterns associated with the VO2peak for each subgroup. The y-axis represents the importance of contribution of each metabolite to the multivariate association with the VO2peak, expressed as the negative logarithm of relative frequencies of permutation-obtained rank products below measured rank products. The x-axis illustrates the direction and strength of bivariate associations between the VO2peak and metabolites, expressed as partial Pearson correlation coefficients of Van der Waerden-transformed variables. The classification of metabolites to major metabolic pathways is color-coded. The mean of the root mean square error (RMSE) based on the test samples was not always higher in the permutations when additionally controlling for phenotypical and clinical variables. Therefore, the respective results from multivariate association analyses (F**/M**) have to be interpreted with caution.
