# Supplementary material for: Sex-Specific Relationship between the Cardiorespiratory Fitness and Plasma Metabolite Patterns in Healthy Humans—Results of the KarMeN Study
Source: Metabolites. 2021 Jul 17;11(7):463. doi: 10.3390/metabo11070463 (PMC8303204; doi:10.3390/metabo11070463)
Supplement: Supplementary file 1 [file metabolites-11-00463-s001.zip › File S3_Classification of metabolites with significant bivariate correlations to metabolic pathways.pdf]

File S3: Classification of metabolites with significant bivariate correlations to metabolic pathways

1. Confounder-adjusted correlations in females (F*)

Classification of Plasma Metabolites Correlating with the CRF to Major Metabolic Pathways (F*)

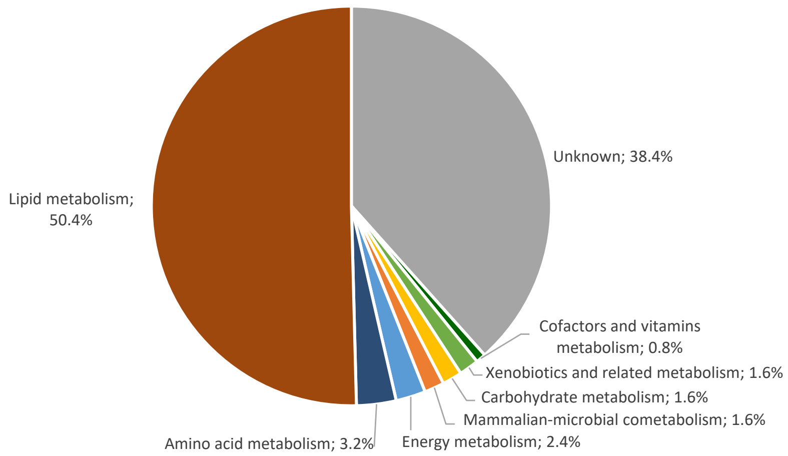

A) Lipid metabolism (50.4%) - Sub-Pathways

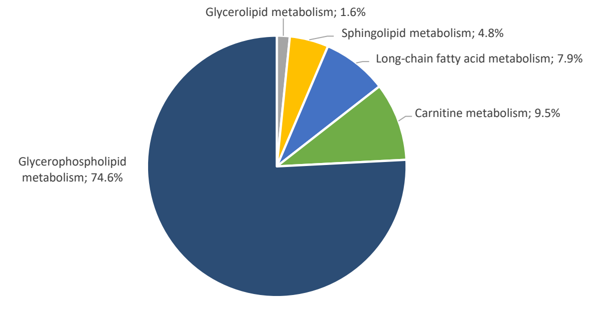

B) Amino acid metabolism (3.2%) - Sub-Pathways

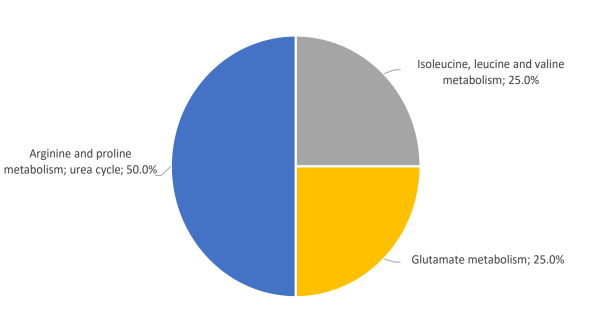

C) Energy metabolism (2.4%) - Sub-Pathways

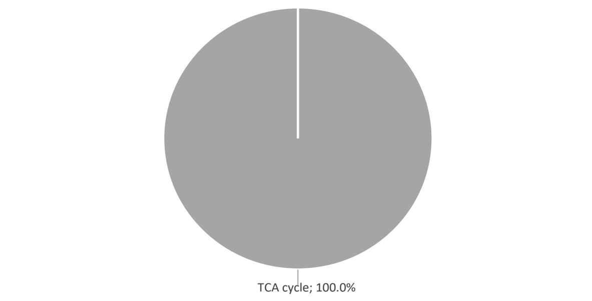

D) Mammalian-microbial cometabolism (1.6%) - Sub-Pathways

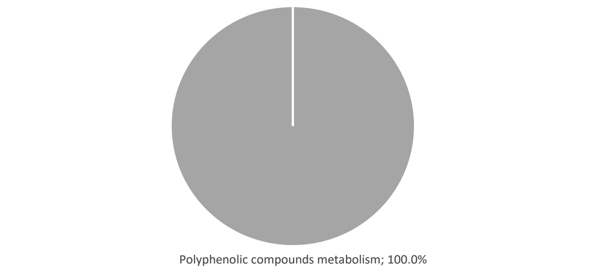

E) Carbohydrate metabolism (1.6%) - Sub-Pathways

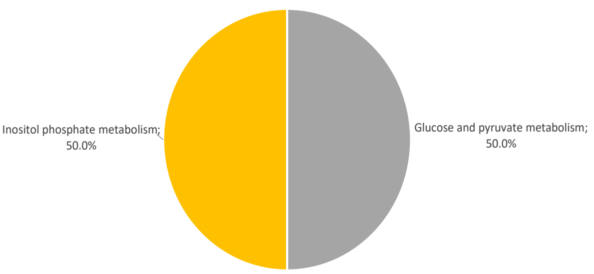

F) Xenobiotics and related metabolism (1.6%) - Sub-Pathways

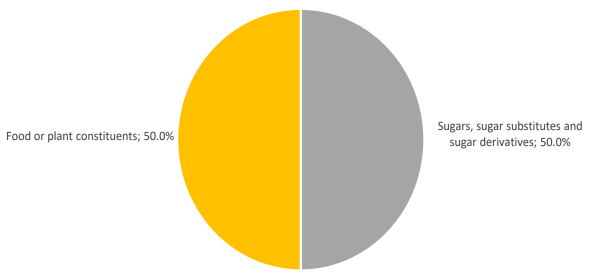

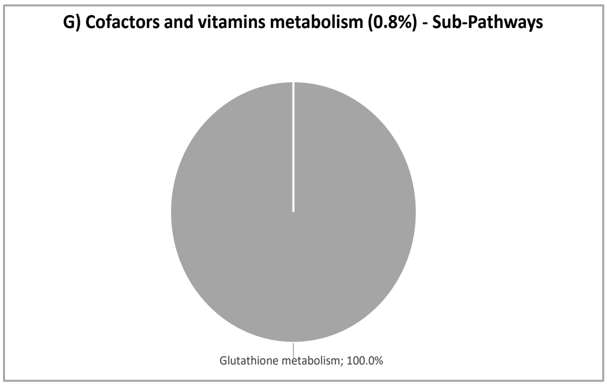

Figure 1. Classification of CRF-correlated metabolites to major metabolic pathways and sub-pathways (females).
125 plasma metabolites showed signficant bivariate correlations with the CRF in females (after adjusting for confounders). Most of them belong to lipid metabolism (63/125) and amino acid metabolism (4/125), followed by energy metabolism (3/125) and mammalian-microbial, carbohydrate or xenobiotics-related metabolism (each 2/125) and cofactors and vitamins metabolism (1/125). 48 plasma analytes were unknown.

2. Confounder- and clinical/phenotypical variables-adjusted correlations in females (F**)

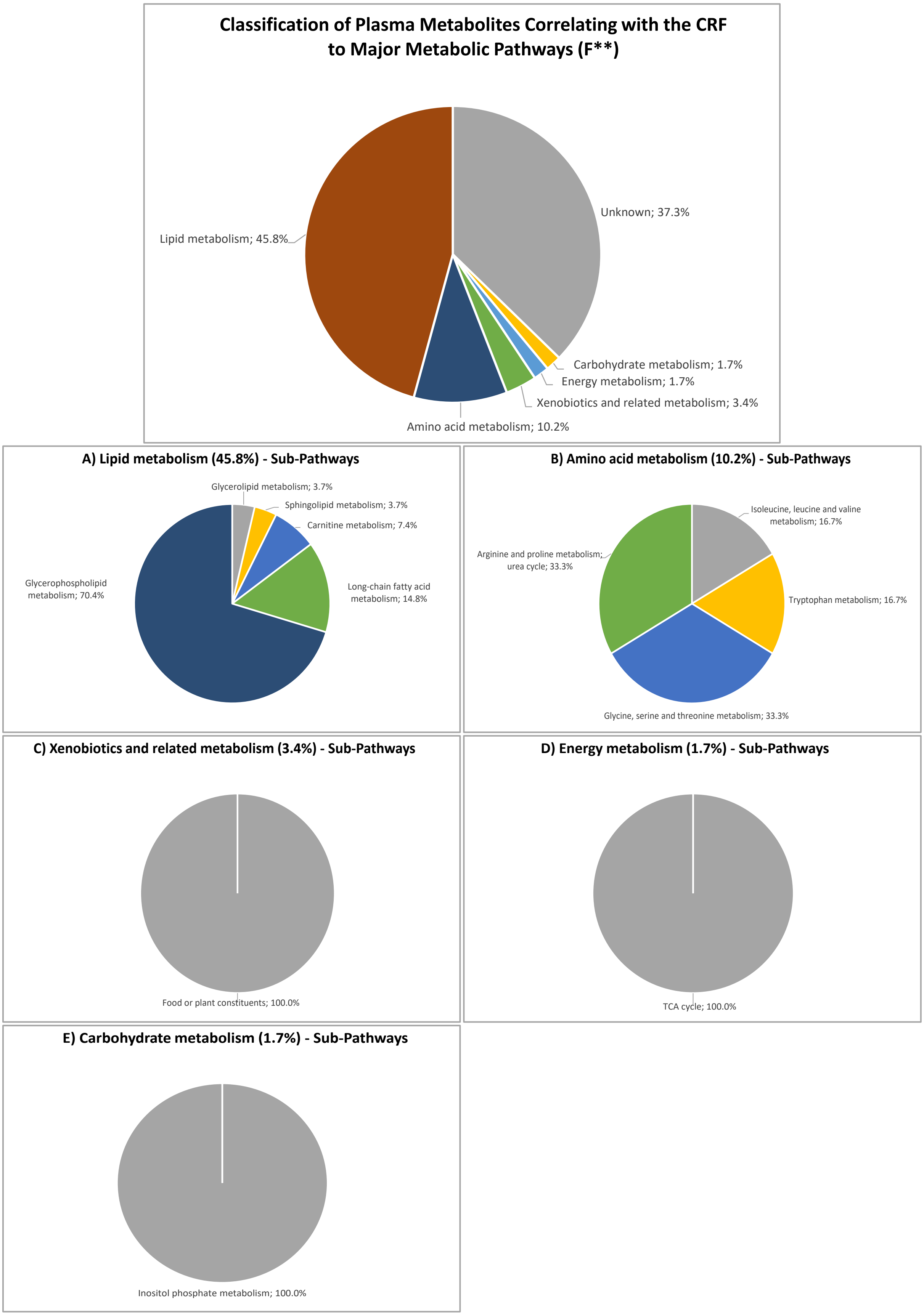

Figure 2. Classification of CRF-correlated metabolites to major metabolic pathways and sub-pathways (females).
59 plasma metabolites showed significant bivariate correlations with the CRF in females (after adjusting for confounders and phenotypical/clinical variables). Most of them belong to lipid metabolism (27/59) and amino acid metabolism (6/59), followed by xenobiotics and related metabolism (2/59), energy or carbohydrate metabolism (each 1/59). 22 plasma analytes were unknown.

3. Confounder-adjusted correlations in males (M*)

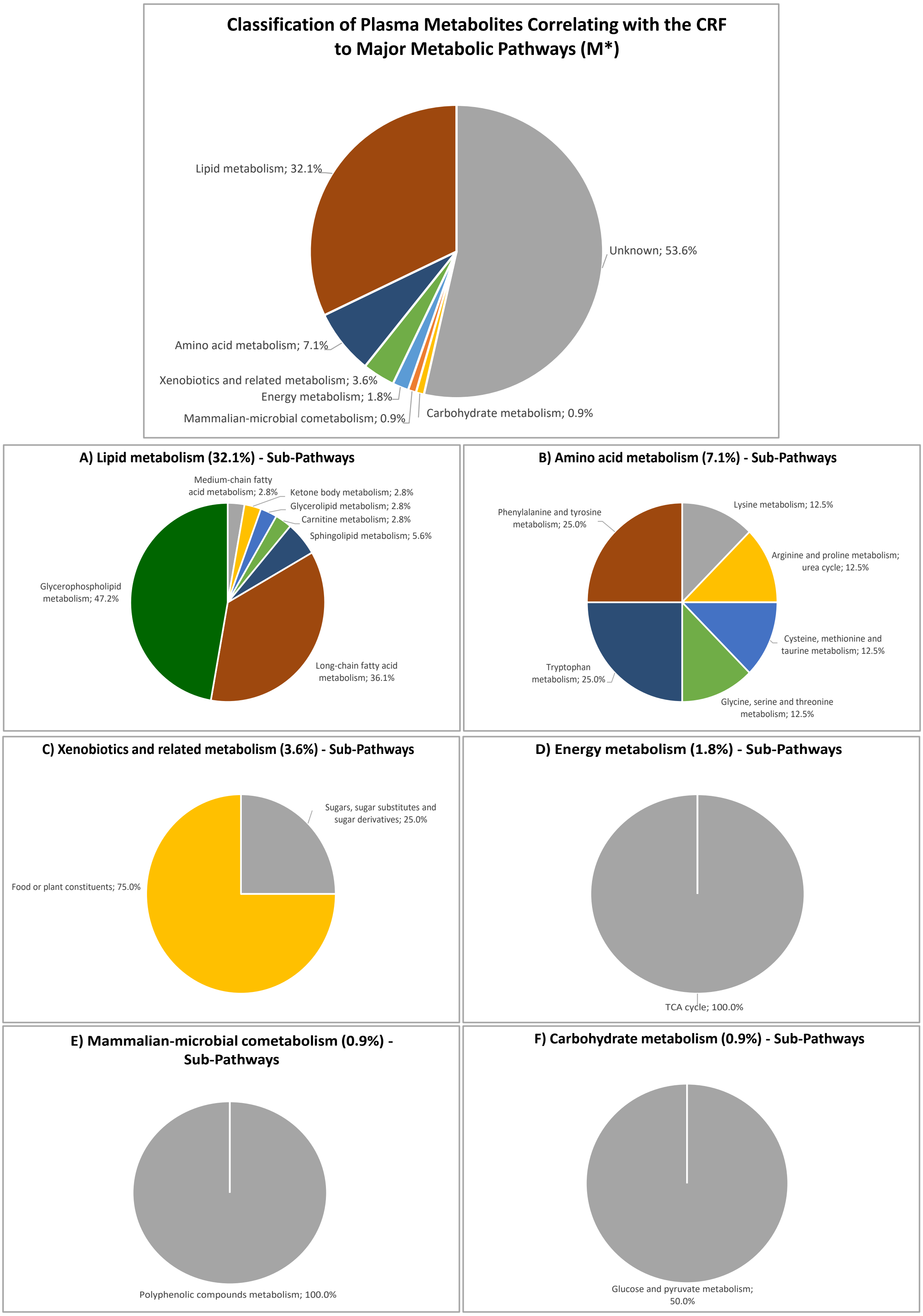

Figure 3. Classification of CRF-correlated metabolites to major metabolic pathways and sub-pathways (males).
112 plasma metabolites showed significant bivariate correlations with the CRF in males (after adjusting for confounders). Most of them belong to lipid metabolism (36/112) and amino acid metabolism (8/112), followed by xenobiotics and related metabolism (4/112), energy metabolism (2/112) and carbohydrate or mammalian-microbial (co-)metabolism (each 1/112). 60 plasma analytes were unknown.

4. Confounder- and clinical/phenotypical variables-adjusted correlations in males (M**)

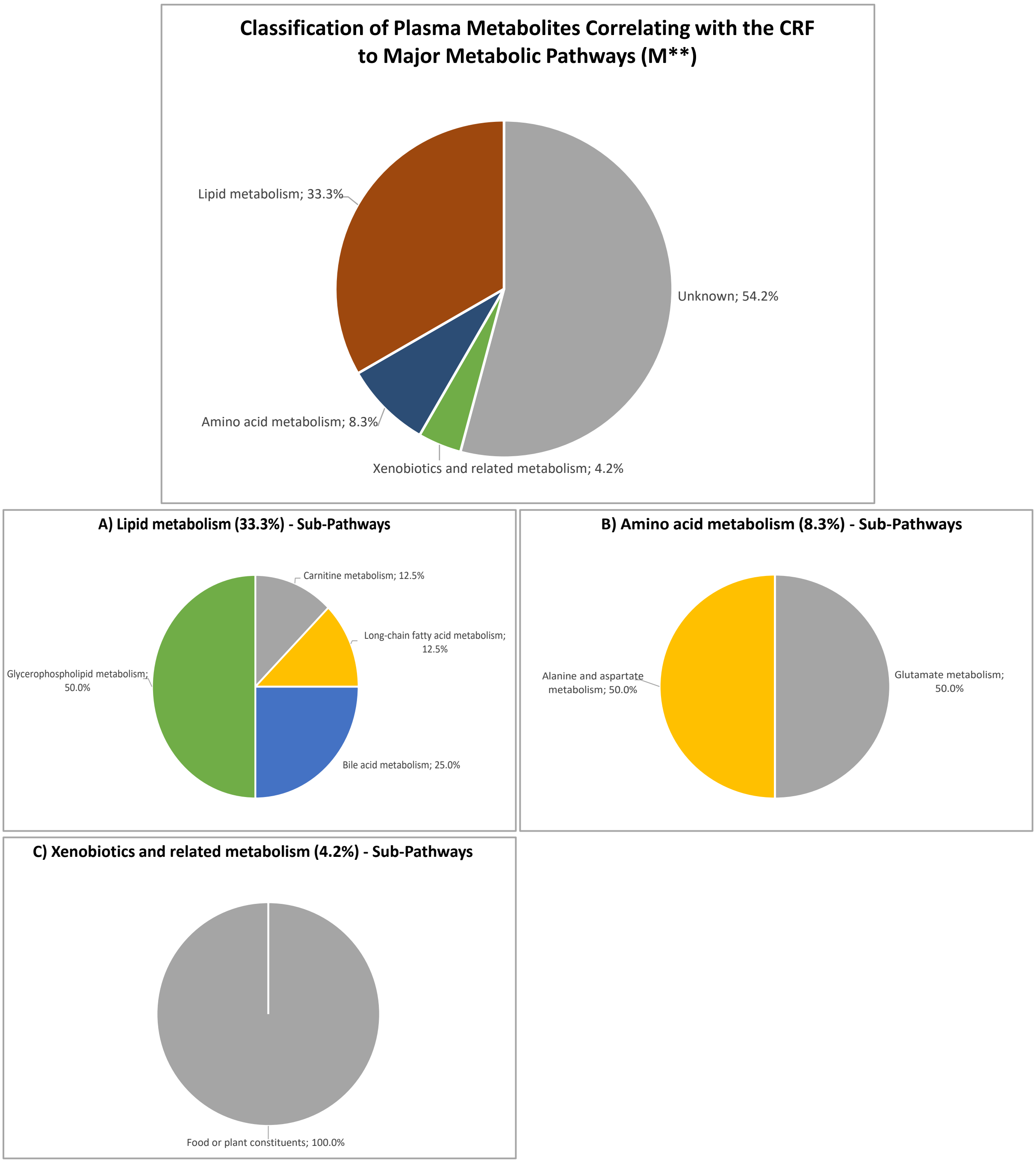

Figure 4. Classification of CRF-correlated metabolites to major metabolic pathways and sub-pathways (males).
24 plasma metabolites showed significant bivariate correlations with the CRF in males (after adjusting for confounders and phenotypical/clinical variables). Most of them belong to lipid metabolism (8/24) and amino acid metabolism (2/24), followed by xenobiotics and related metabolism (1/24). 13 plasma analytes were unknown.
